# Approximate simulation of cortical microtubule models using dynamical graph grammars

**DOI:** 10.1088/1478-3975/acdbfb

**Published:** 2023-07-07

**Authors:** Eric Medwedeff, Eric Mjolsness

**Affiliations:** 1 Computational Science Research Center, San Diego State University, 5500 Campanile Drive, San Diego, CA 92182, United States of America; 2 Department of Computer Science, University California Irvine, Irvine, CA 92697-3435, United States of America; 3 Department of Mathematics, University California Irvine, Irvine, CA 92697-3875, United States of America

**Keywords:** numerical computing, dynamic graph grammars, cortical microtubule array, simulation algorithm, cell complex, local graph dynamics, operator splitting

## Abstract

Dynamical graph grammars (DGGs) are capable of modeling and simulating the dynamics of the cortical microtubule array (CMA) in plant cells by using an exact simulation algorithm derived from a master equation; however, the exact method is slow for large systems. We present preliminary work on an approximate simulation algorithm that is compatible with the DGG formalism. The approximate simulation algorithm uses a spatial decomposition of the domain at the level of the system’s time-evolution operator, to gain efficiency at the cost of some reactions firing out of order, which may introduce errors. The decomposition is more coarsely partitioned by effective dimension (d=0 to 2 or 0 to 3), to promote exact parallelism between different subdomains within a dimension, where most computing will happen, and to confine errors to the interactions between adjacent subdomains of different effective dimensions. To demonstrate these principles we implement a prototype simulator, and run three simple experiments using a DGG for testing the viability of simulating the CMA. We find evidence indicating the initial formulation of the approximate algorithm is substantially faster than the exact algorithm, and one experiment leads to network formation in the long-time behavior, whereas another leads to a long-time behavior of local alignment.

## 1. Introduction and background

### 1.1. Overview

Dynamic graphs are graphs that change over time and are capable of encoding the changing state of complex systems. Graphs with local dynamics provide a mathematical framework for understanding changing relationships between objects. We can formulate and enable the use of dynamic graphs by providing a high level language for their dynamics. Dynamical graph grammars (DGGs) [1] allow for an expressive and powerful way to declare a set of local rules to model a complex, dynamic system with graphs. The DGG formalism is flexible for modeling and allows a wide array of models to be created in the natural sciences.

DGGs have well-defined meaning. They map graph dynamics into a master equation, a set of first order linear differential equations governing the time evolution of joint probability distributions of state variables of a dynamic system. Using operator algebra [2], DGGs can be simulated using an exact algorithm that subsumes Gillespie’s stochastic simulation algorithm (SSA) [3], which is closely related to Kinetic Monte Carlo algorithms of statistical physics [4]. As does the SSA, the exact algorithm becomes slow for large systems. Using operator splitting, a faster approximate algorithm for spatially embedded graphs can be derived. In section 2 we discuss the preliminary work done on the approximate algorithm, and areas for improvement.

In this work we present a paradigm for model creation and demonstrate the utility provided by DGGs. In particular, we focus on one specific example in biology, plant cell division, and one particular system in that process, the cortical microtubule array (CMA). We further restrict our graphs, and require them to be spatially embedded in Euclidean space.

### 1.2. Biological motivation

Eukaryotic organisms comprise complex cells with many subsystems that are well suited to be modeled with dynamic graphs. Over time cells can divide allowing for a plant to grow, among other processes. Understanding the exact biomechanical mechanisms taking place during cell division in plants is a long-standing question [5], but it is known that there is a connection between division plane orientation in plants and a change in the orientation of cortical microtubules (MTs) associated with the plasma membrane [6], as they form the pre-prophase band (PPB). For example, one hypothesis for the PPB orientation process is ‘survival of the aligned’ [7], and another is alignment through selective katanin mediated severing [8]. The ensemble of MTs associated with the plasma membrane of the cell is the CMA. The question of how MTs contribute to cell shape and other processes during cell division motivated us to develop a simplified model for the dynamics found in the CMA, with potential to extend this work to larger systems with more complicated dynamics and interacting networks at different spatial scales.

### 1.3. Stochastic chemical kinetics

The SSA is an example of *stochastic* modeling, as opposed to the *deterministic* modeling approach [9]. In a deterministic approach, the time-continuous processes are wholly predictable and can be governed by the *reaction-rate equations* [10], a set of coupled ordinary differential equations (ODEs). The stochastic approach is a type of random-walk process that is completely encoded in the *master equation*. The master equation is itself a high dimensional linear differential equation, governing the rate at which probability flows through different states in the system. However, systems can become very large due to an exponential state space explosion with respect to the number of biological variables, and the systems may have infinite dimensional state spaces, making the analytical solution to the master equation computationally intractable or impossible.

The work of Gillespie [11] uses the Monte Carlo method and kinetic theory to rigorously derive the exact SSA for chemical kinetics. The derivation makes a case based on several assumptions about the systems, the most important being the system contains a large number of well-mixed molecules at thermal equilibrium. After making key assumptions, it is necessary to set reaction rates—which can be difficult to determine. Three routes for determining rates are lab measurements, giant *ab initio* quantum mechanical calculations or machine-learning generalizations therefrom, and parameter optimizations in the context of system-level observations together with the use of other known reactions rates that are more easily measurable. Finally, an event is sampled from a conditional density function. The Monte Carlo procedure does not give the analytic solution to the master equation, but it does yield an unbiased sample trajectory of a system. It effectively provides a *realization* by means of numerical simulation.

As powerful as the exact SSA is, it is prohibitively slow, since each reaction event must be computed in order. Numerous methods have been proposed to speed up the exact SSA. τ-Leap [12] fires all reactions in a window of τ before updating propensity functions, saving computation at the cost of errors. Later, it was made even more efficient [13]. R-Leaping [14] lets a preselected number of reactions fire in a simulation step, again at some cost in accuracy. The Exact R-Leap, ‘*ER*-Leap’ [15] modifies the R-Leaping algorithm to both be exact and provide a substantial speed up over SSA. *ER*-leap was later improved upon and parallelized in *HiER*-Leap [16]. More recently, *S*-Leap [17] was introduced as an adaptive, accelerated method that bridges the methods of τ-Leaping and R-Leaping. There are many other works on speeding up the original SSA as well.

Our work builds on this rich history and complements it. We are not just interested in solving the master equation for stochastic chemical kinetics. Instead, we’d like to solve a broader class of problems in biology and beyond, by representing the dynamics of spatially extended objects using graphs. The foundational work for the mathematical theory will be briefly discussed in the DGG formalism section and the curious reader may refer to [1, 2, 18] for more detailed information.

### 1.4. Graph theory

We will use the following graph theory and notation. A *graph* (*undirected*) G=(V,E) is a set of V vertices and set of edges E⊆{{u,v}∣u,v∈V}, each an unordered pair of V, where the elements u,v∈V are vertices. V(G) is the set of vertices of graph G and E(G) is the set of edges of graph G. Now, let G=(V,E) and $G^{\prime }=\left(V^{\prime },E^{\prime }\right)$. The graph G is homomorphic to $G^{\prime }$ if there exists a mapping $f:V⟶V^{\prime }$ that preserves the adjacency of vertices i.e. $\left(v,v^{\prime }\right)\in E⟹\left(f(v),f\left(v^{\prime }\right)\right)\in E^{\prime }$. We call this a *homomorphism* from G to $G^{\prime }$. If the function f is bijective and its inverse is a homomorphism, then f is also an [19] *isomorphism*.

A *labeled graph* G is defined as G=(V,E,α), where (V,E) is a graph and α:V⟶L is a function assigning labels to vertices. To each labeled graph there corresponds an (unlabeled) graph (V,E) without the labels. We define a *label-preserving homomorphism* of labeled graphs to be a graph homomorphism that preserves the labels exactly, without remapping them. We define a *match* to be an injective label-preserving graph homomorphism $G↪G^{\prime }$. Informally, a match locates a ‘copy’ of G as a subgraph inside of $G^{\prime }$ for which vertices, edges, and labels are all preserved.

**Figure 1.**
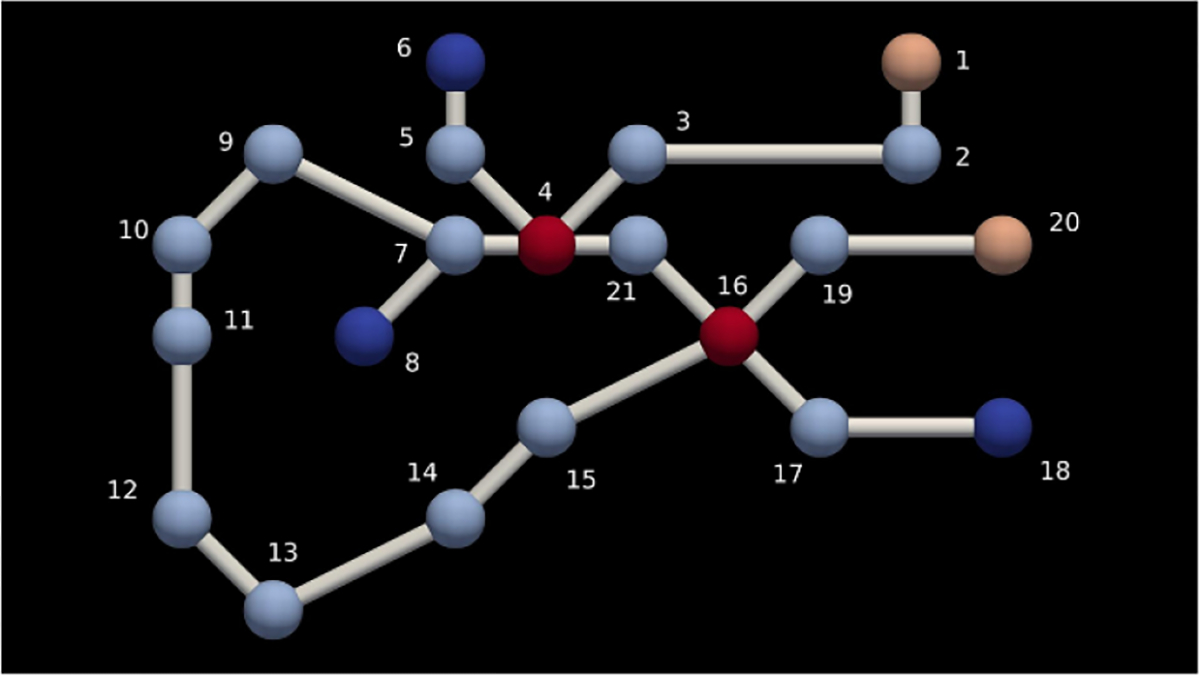
A visual example of a graph labeled by number and color.

A labeled graph can be seen in figure 1. Here, the nodes are uniquely labeled using positive integers and the edges remain unlabeled. The discrete vertex labels have been mapped to a color set and visualized with those colors. In this case, the graph has no spatial embedding, so we could visualize the graph in many different ways.

A *dynamic graph*, G(t) is a graph that changes over time. The change can either be in the form of vertex/edge creation or destruction, or the change of label parameters. Mathematically, we write $G(t)=\left(V(t),E(t),{\;\alpha }_{t}\right)$, where $\alpha_{t}\;:V(t)⟶L$.

### 1.5. Extended objects and the expanded cell complex (ECC)

Declarative modeling of complex biological systems requires a way to describe non point-like *extended objects* [1]. Examples of extended objects are polymer networks in the cytoskeleton, and multi-cellular tissues. In this section, we use a mix of standard and non-standard definitions used in construction of extended objects and we introduce the ‘ECC’ used in the approximate simulation algorithm.

Graphs augmented with labels are expressive mathematical objects capable of a high level of abstraction, and we use these for the representations of all of our extended objects. As defined in [1], *numbered graphs* are special cases of labeled graphs that have unique consecutive non-negative integer labels for vertices. If the graph in figure 1 did not have colors assigned to nodes it would be a numbered graph. A *graded graph*, on the other hand, is a graph where vertices are labeled non-uniquely with a level number, associated with spatial resolution which can only differ by {0, ±1} between neighbors. A *stratified graph* labels vertices by a non-negative integer ‘dimension’ of the stratum to which they belong. *Graded stratified graphs* have both dimension and level number vertex labels with suitable constraints.

A special case of stratified graphs is the *abstract cell complex*. The abstract cell complex is a graph that is used to represent the topology of a space in the manner of a CW cell complex [20]. It has further constraints on the dimension labels. A *graded abstract cell complex* can represent topological properties of the space with the addition of level numbers associated with spatial resolution.

**Figure 2.**
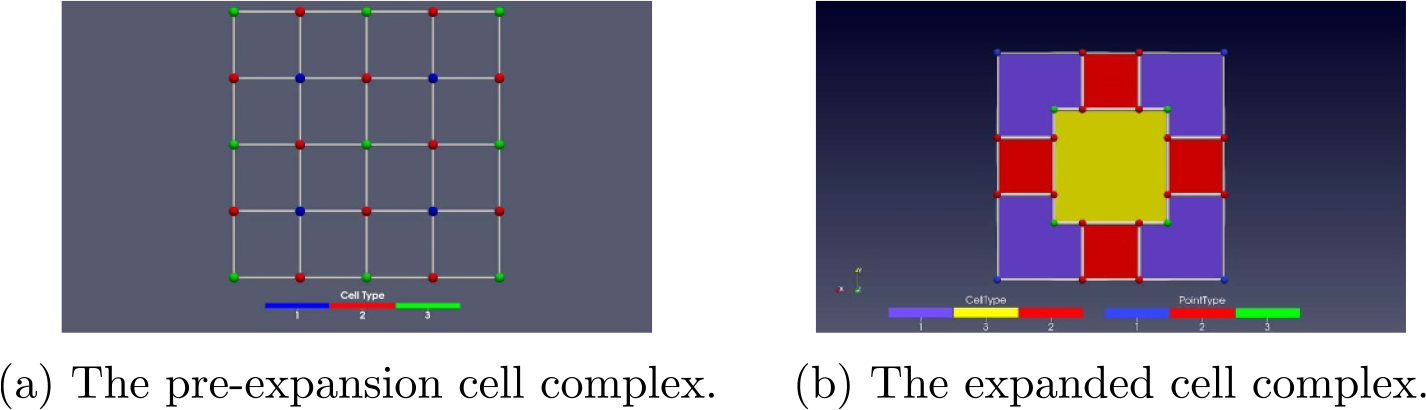
Expanding the cell complex of a 4 × 4 Cartesian grid into well-separated lower dimensional cells. For this example, only the interior is expanded.

‘*Expanding*’ is a process of consistently mapping each lower dimensional cell in a cell complex to a cell of the highest dimension in a new ‘expanded’ cell complex. By expanding as in [21, 22] all of the abstract cell complex cells with dimension numbers less than the maximum, we get an ECC. We only apply expansion to the interior of lower dimensional cells. In our case we apply expansion to a two level graded abstract cell complex with a coarse scale 2D grid that is refined to a finer scale 2D grid. Figure 2 exhibits a side by side visualization of a pre-expansion (figure 2(a)) cell complex and its post expansion (figure 2(b)) cell complex. In figure 2(b), lower dimension interior cells are expanded such that they always have a ‘collar’ width less than the cell of the dimension above, so that cells of the same dimension are always separated by at least a dimension-specific minimum distance. This key criteria of the ECC is *well-separatedness*, as will be explained more in the methods section. The ‘collar’ we refer to is related to the idea of a tubular neighborhood in differential topology [23]. Further discussion of cell complex theory can be found in [20, 24–27].

### 1.6. Related work

There already has been work done to simulate plant MT dynamics [6, 28]. However, there is no other work to our knowledge that does it with dynamic graph grammars. The tool Plenum [29] implements DGGs as an embedded symbolic meta-programming language in Mathematica. However, it is not scalable because it uses the exact algorithm—which is only practical for small systems. Additionally, Plenum supports graphs by using unique object identifiers (OIDs), but does not directly support graphs as a native data structure. Improvements to the algorithm used in Plenum and a scalable solution is necessary to modernize the current work and allow for faster and more realistic results.

## 2. Methods

### 2.1. DGGs formalism

DGGs are a further refinement of the Dynamical Grammars (DGs) [18], which generalized Stochastic Parameterized Grammars (SPGs) [18] by the inclusion of differential equation rules. SPGs function to unify the formalism of generative grammars, stochastic processes, and dynamic systems. While SPGs can be applied to graphs, DGGs include all the related formalisims of SPGs and DGs, along with an additional and expressive modeling language framework for graphs. The semantics of the DGG formalism starts with DGG models $M_{DGG}$ in language $L_{DGG}$ and using a compositional map $\Psi_{DGG}$, maps the declarative grammar rules in the model to a valid dynamical system expressed by a master equation.

The master equation represents the time evolution of a continuous time Markov process. It can be written in the form:

(1) $$P^{\prime }(t)=W\cdot P(t),$$

with the equation having the formal (but usually not practical) solution:

(2) $$P(t)=e^{tW}\cdot P(0).$$

W is called the model system’s ‘time-evolution operator’, since it entirely specifies (in a probabilistic way, which can specialize to deterministic dynamics if need be) how the model evolves in time.

Let Ψ(M)=W(M) be a semantic map over DGG models comprising rules indexed by r, and ${\overset{ˆ}{W}}_{r}\equiv {\overset{ˆ}{W}}_{{LHS}_{r}\to {RHS}_{r}}$ be an operator that specifies the non-negative flow of probability between states under each rule r. Then Ψ is ‘compositional’ if it sums the operators $W_{r}$ over rules thusly:

(3a) $$W=\sum_{r}W_{r}$$

(3b) $$W_{r}\equiv {\overset{ˆ}{W}}_{r}-D_{r}$$

(3c) $$D_{r}\equiv \text{diag}(1\cdot {\overset{ˆ}{W}}_{r})$$

where equation (3a) states that rule operators sum up, equation (3b) states rules conserve probability, and equation (3c) represents the summed conditional probability outflow per state. The operators for rules indexed by r∈M map to the operator sum and the dynamics can be defined under the ME.

Parameterized grammar rules extend the pure reaction rules to an additional parameterized space and allow for a more expressive form of modeling. This gives rise to the SPG. The probability space for the SPG was defined in [29]. A form of a stochastic parameterized rule is:

(4) $${\left\{\tau_{\alpha \left(p\right)}\left[x_{p}\right]|p\in L_{r}\right\}}_{*}⟶{\left\{\tau_{\beta \left(q\right)}\left[x_{q}\right]|q\in R_{r}\right\}}_{*}\;\;\mathrm{with}\;\rho_{r}\left(\left[x_{p}\right],\left[y_{q}\right]\right)$$

where $\tau_{\alpha (p)}\left[x_{p}\right]$ and $\tau_{\beta (q)}\left[x_{q}\right]$ are the object types parameterized by parameters $x_{p}$ and $x_{q}$. Note that $x_{p}$ and $x_{q}$ may be vectors. Again, r is the rule index, and $L_{r},\;R_{r}$ are the left and right hand side argument list indexed sets. So, p and q represent the position of $\tau_{\alpha (p)}\left[x_{p}\right]$ and $\tau_{\beta (q)}\left[x_{q}\right]$ in their respective argument lists. Finally, $\rho_{r}\left(\left[x_{p}\right],\left[y_{q}\right]\right)$ is the reaction rate function of both the incoming and outgoing parameters. $\rho_{r}\left(\left[x_{p}\right],\left[y_{q}\right]\right)⟶R^{+}$ is a non-negative propensity rate function. If $\rho_{r}\left(\left[x_{p}\right],\;\left[y_{q}\right]\right)$ is integrable over output parameters it can be decomposed into a rate function over input parameters and a conditional probability over the output parameters:

(5) $$\;\rho_{r}\left(\left[x_{p}\right]\right)\;\equiv \int \;\rho_{r}\left(\left[x_{p}\right],\left[y_{q}\right]\right)\;\Delta \left[y_{q}\right]\;\;P\left(\left[y_{q}\right]|\left[x_{p}\right]\right)\;\equiv \frac{\rho_{r}\left(\left[x_{p}\right],\;\left[y_{q}\right]\right)}{\rho_{r}\left(\left[x_{p}\right]\right)}\;\;\rho_{r}\left(\left[x_{p}\right],\left[y_{q}\right]\right)\;\equiv \rho_{r}\left(\left[x_{p}\right]\right)*P\left(\left[y_{q}\right]|\left[x_{q}\right]\right).$$

For clarity, grammar rules will be written decomposed in this manner.

Parameterized rules are encoded into the master equation and we can derive a simulation algorithm [2]. We can elevate these parameterized rules to include graphs by adding unique discrete object IDs [30] as parameters.

Using operator algebra [2] we can derive an exact time warping simulation algorithm [2] (algorithm 1), and add in differential equation rules:

(6) $${\left\{\tau_{\alpha \left(p\right)}\left[x_{p}\right]|p\in L_{r}=R_{r}\right\}}_{*}⟶{\left\{\tau_{\beta \left(q\right)}\left[x_{q}\right]|q\in R_{r}=L_{r}\right\}}_{*}\;\;\mathrm{solving}\;\{\frac{dx_{p,j}}{\;dt}=v_{p,j}\left(\left[x_{k}\right]\right)|p,j\}.$$

Here, everything remains the same in regard to notation, except the left hand side (LHS) and right hand side do not change in number or object type, but the parameters can evolve by solving a differential equation. When we combine these differential rules with the parameterized rules, we get DGs [16].

The exact algorithm simulates a single trajectory of a continuous time stochastic process. Propensity functions are factored into a product of the rate function and a distribution of output parameters conditioned on input parameters, as in equation (5). While the simulation time is less than the maximum, we compute the time until the next reaction and modify the system when it occurs. The process is very similar to the standard SSA, but with the inclusion of the time warping equation. The warp equation is an ODE to keep track of the time until the next event and must be solved as part of the system of ODEs governing the time evolution of the parameters. When a reaction does occur, the state of the system is modified according to the rule instance selected and the parameters are sampled from the conditional distribution of the factored propensity function.


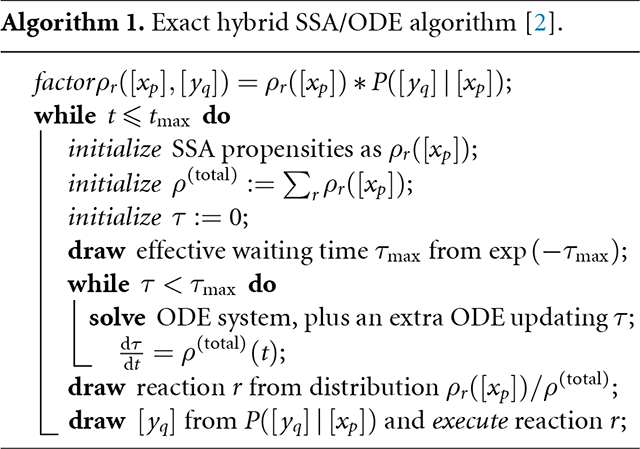


DGs [30] are able to handle graph representations using unique OID parameters. However, using OIDs to represent graphs decreases readability and natural expressiveness. Alternatively, using a formal graph notation [1], DGGs can be represented using a different form:

(7) $$G\langle \langle \lambda \rangle \rangle ⟶G^{\prime }\left\langle \left\langle \lambda^{\prime }\right\rangle \right\rangle \;\mathrm{with}\;\;\rho_{r}\;\text{or}\;\mathrm{solving}\;\dot{x}=v\text{.}$$

Here G⟨⟨λ⟩⟩ is the LHS labeled graph with label vector λ and $G^{\prime }\left\langle \left\langle \lambda^{\prime }\right\rangle \right\rangle$ is the right hand side labeled graph with label vector $\lambda^{\prime }.\;G$ and $G^{\prime }$ without their label vectors λ and $\lambda^{\prime }$ are numbered graphs, so that the assignment of label component $\lambda_{i}$ to graph node member i is unambiguously specified. We have the usual ‘solving’ and ‘with’ clauses. For examples of such graph grammar rules, see the supplementary material.

### 2.2. Approximating the exact simulation algorithm

The forgoing exact algorithm is powerful and works for multiple rules of different forms [2]; however, it is prohibitively slow for large systems. A single run of the exact algorithm yields only one trajectory. In practice thousands or more may be needed to be run to compute meaningful statistics or to recover outcome density functions. The type of rules we use are expressed as graphs [1] and extend previous work [30] by being a more efficient and readable representation of DGG rules compared to using the OIDs mentioned in the previous section.

We make two key assumptions in our approximation of the exact algorithm: spatial locality of the rules and well-separatedness of the cell complex used to decompose biological space into domains. Consider the spatial locality constraint for graphs. The system state comprises extended objects taking the form of labeled graphs. Each of the nodes in a graph is labeled with a vector-valued position parameter. Additional parameters are allowed and have no spatial constraint. Our graph grammar rules are made spatially local by virtue of their propensity functions. We use spatial locality to define local neighborhoods of rule firings. Any rule instantiations outside this neighborhood have zero or near zero propensity that decreases rapidly, for example exponentially, with distance. Hence, any two objects in the system that are too far apart have a very small chance of reacting, and their potential interactions are ignored.

Spatial locality also allows us to decompose the domain of the simulation space into smaller, well-separated geometric cells. In the context of the simulation algorithm, a ‘cell’ refers to a computational spatial domain, which differs from the biological notion of a cell. Such a geometric cell (geocell) is a cell of an ECC, labeled by the dimension of the corresponding cell in the unexpanded cell complex. Lower dimensional cells of the cell complex are expanded to be large enough to keep rule instances from spanning multiple same-dimensional geocells. An example can be seen in figure 2. By setting these geocells to be large enough (at least several factors larger than the exponential ‘fall off distance’), we are able to logically map rule instances to well-separated geocells.


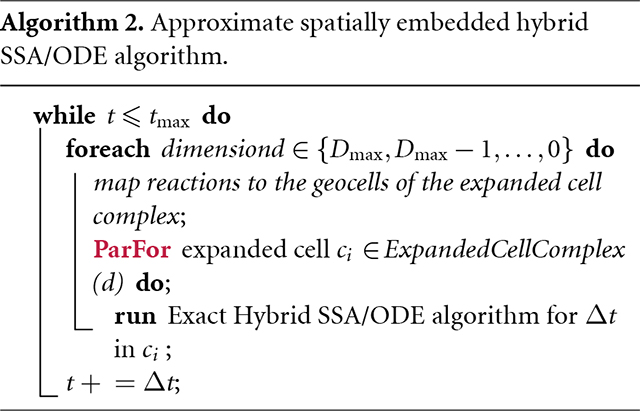


The operator W in equation (1) assumes a state space and specifies the probability flow on that space for all of the extended objects in our system. Considering equation (3a), $W=\sum W_{r}$, the method we propose to approximate $e^{tW}$ is an operator splitting algorithm that imposes a domain decomposition by means of an ECC that corresponds to summing operators, $W=\sum_{\left(d\right)}\;W_{\left(d\right)}=\sum_{\left(d,c\right)}\;W_{\left(c,d\right)}$, over pre-expansion dimensions d, and cells c of each dimension:

(8a) $$e^{tW}\;\approx {\left(\prod_{d↓}e^{\frac{t}{n}W_{\left(d\right)}}\right)}^{n\to \infty }$$

(8b) $$e^{t^{\prime }W_{\left(d\right)}}\;=\prod_{c\subset d}e^{t^{\prime }W_{\left(c,d\right)}}\;\text{where}\;\left[W_{\left(c,d\right)},W_{\left(c^{\prime },d\right)}\right]\approx 0\;\;\text{and}\;t^{\prime }\;\equiv \frac{t}{n}$$

(8c) $$W_{\left(c,d\right)}\;=\sum_{r}W_{r,c}\equiv \sum_{r}\sum_{\left\{\begin{array}{c} R|\varphi \left(R\right)=c,\\ R\;\text{instantiates}\;r \end{array}\right\}}W_{r}\left(R|c,d\right).$$

Equation (8a) is a first-order operator splitting, by solution phases of fixed cell dimension, where d↓ means we multiply from right to left in order of highest dimension to lowest. It incurs an approximate error of $O((t/n{)}^{2})$. Equation (8b) uses the fact that the resulting cells c of fixed dimension d are all well-separated geometrically with enough margin (due to the ‘collar’ of dimension $d^{\prime }\neq d$ [21]) so that reaction instances $R,R^{\prime }$ commute to high accuracy if they are assigned to different cells $c,c^{\prime }$ of the same dimensionality, by some reaction instance allocation function φ. The commutators of equation (8b) can be calculated as derived in [31], but they will inherit the exponential falloff with separation that we assumed for the rule propensities (see [31], equation 12 therein). Hence, the dynamics $e^{tW_{\left(c,d\right)}}$ of different cells $c,c^{\prime }$ of the same original dimension d and can be simulated in any order, or in parallel, at little cost in accuracy.

The operator splitting and the function φ introduce a major opportunity for parallel computing, because the exponentials defined in each cell c of a given dimensionality d can all be sampled independently of one another. This potential parallelism includes the possibly heavy computation of solving ODEs specific to cell *c*. Equation (8c) then defines the geocell-specific operator for the process to be simulated by algorithm 1 [2], specialized to the case of graphs. The resulting parallel algorithm is outlined in algorithm 2. It can be seen that without domain subdivisions, the approximate algorithm reduces to the exact algorithm. A more complete mathematical treatment of the approximate algorithm using DGG commutators computed as in [31] to bound the operator splitting errors will be the topic of future work.

### 2.3. Developing the prototype DGG simulator

To conduct our experiment with simplified CMA models, we have implemented a prototype DGG simulator in C++ [32]. The simulator is capable of using both the exact and approximate algorithms. The prototype simulator makes use of a dynamic graph library, an ECC, a subgraph-specific pattern recognizer (SSPR), an ODE solver, an input file reader, and an output file generator.

The core data structure in the DGG simulator is the graph. The DGG formalism declaratively specifies what type of graph rewrites can occur, but it does not specify how rewrites are performed or how graphs should be represented on a computer. To address the ‘how’ we have developed our own dynamic graph library, Yet Another Graph Library (YAGL). YAGL provides a dynamic graph data structure, along with rewrite operations. Other functionality has been implemented as needed, and YAGL will continue to develop concurrently with the simulator implementation.

**Figure 3.**
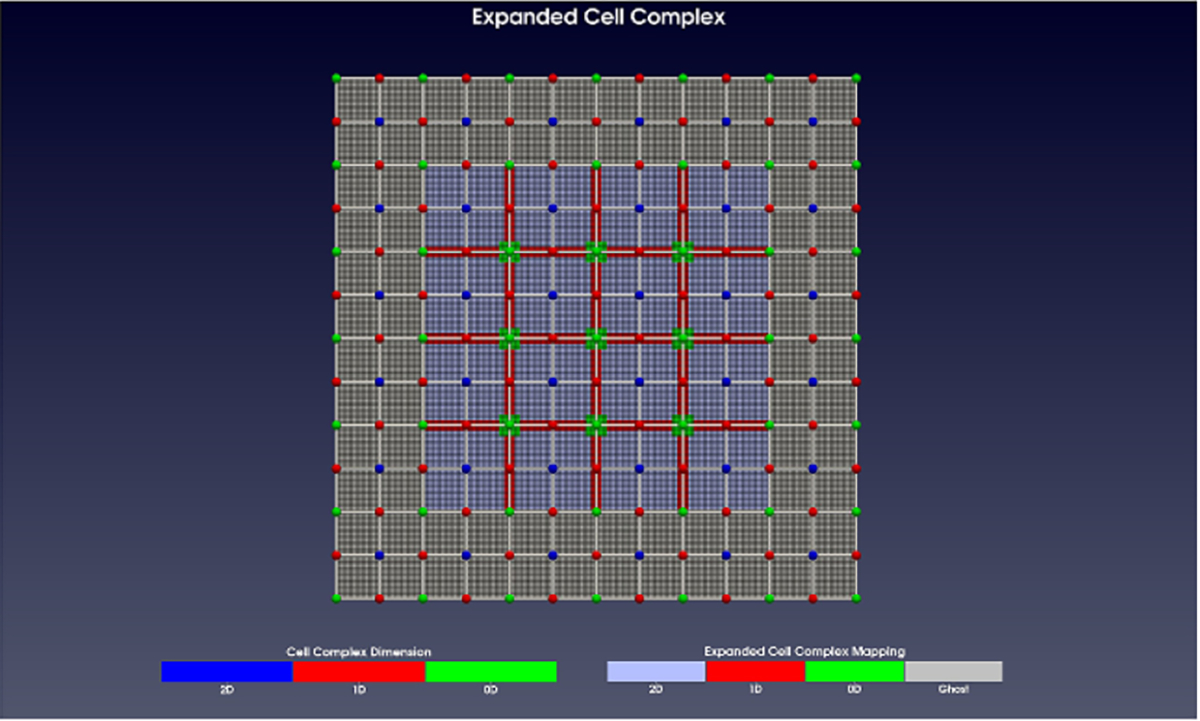
Expanded cell complex (ECC) with well-separated lower dimensions. Regions of the same pre-expansion dimension are separated from each other. Note how only interior lower dimensional cells are expanded. A reaction grid is aligned with the geocells, and reaction cells are smaller than geocells. The outer boundary of the ECC is padded with optional ghosted geocells. Ghosted geocells are just geocells that are not processed by algorithm 2. These optional ghost cells operate as a buffer for any computational errors or as a capture condition in the case of no grammar rules addressing boundary conditions.

The ECC is what the simulator uses to manage the topology and geometry of the simulation space. A spatial reaction grid is used to manage the propensity function ‘fall off’ of a rule instance. The ECC and a reaction grid are seen in figure 3. For this work, a cell complex of a regular Cartesian grid is expanded. A cell complex graph is used for the expansion and it is labeled with additional data for the well-separatedness criteria. The reaction grid is also a Cartesian grid and it is aligned with the ECC. Regions of the same pre-expansion dimension are separated from each other by a minimum distance. Further, reaction grid cells keep reactions spatially local and are smaller than the minimum separation distance of geocells.

We use a SSPR to find all matching LHSs of each grammar rule. For our purposes, a recognizer is a program that identifies all labeled subgraphs that match one to one with a given LHS labeled graph. Subgraph matches are found by using manually written search code for individual LHS rather than general-purpose subgraph isomorphism algorithms [33, 34]. The SSPR implementation we use includes the search code for each individual LHS grammar rule in the model.

To build a SSPR, we need to determine a way to find all the applicable matches of a given LHS grammar rule in the system graph, $G_{SYS}$. We denote the LHS of any rule as $G_{\text{LHS}}$. The system graph is our search space, and the pattern of the LHS is the target. The process of recognizing a single instance of the target graph in the search space is what we mean by *subgraph pattern recognition*. Finding every valid instance of target graph $G_{\text{LHS}}$ is *matching*.

Since it would be prohibitively hard to directly search for all possible functions $f:G_{LHS}⟶G_{SYS}$, where f is an edge preserving map, we need to apply some heuristic filter. Let $G_{T}$ be a rooted spanning tree of target graph $G_{\text{LHS}}$. If $G_{T}$ and f exist, then there must exist some mapping $g:G_{T}⟶G_{SYS}$. We can represent the entire process using the commutative diagram of graph homomorphisms as in equation (9):

(9)

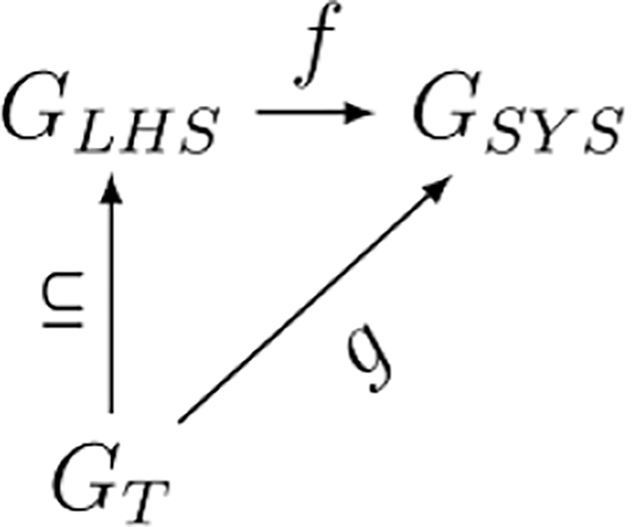



**Figure 4.**
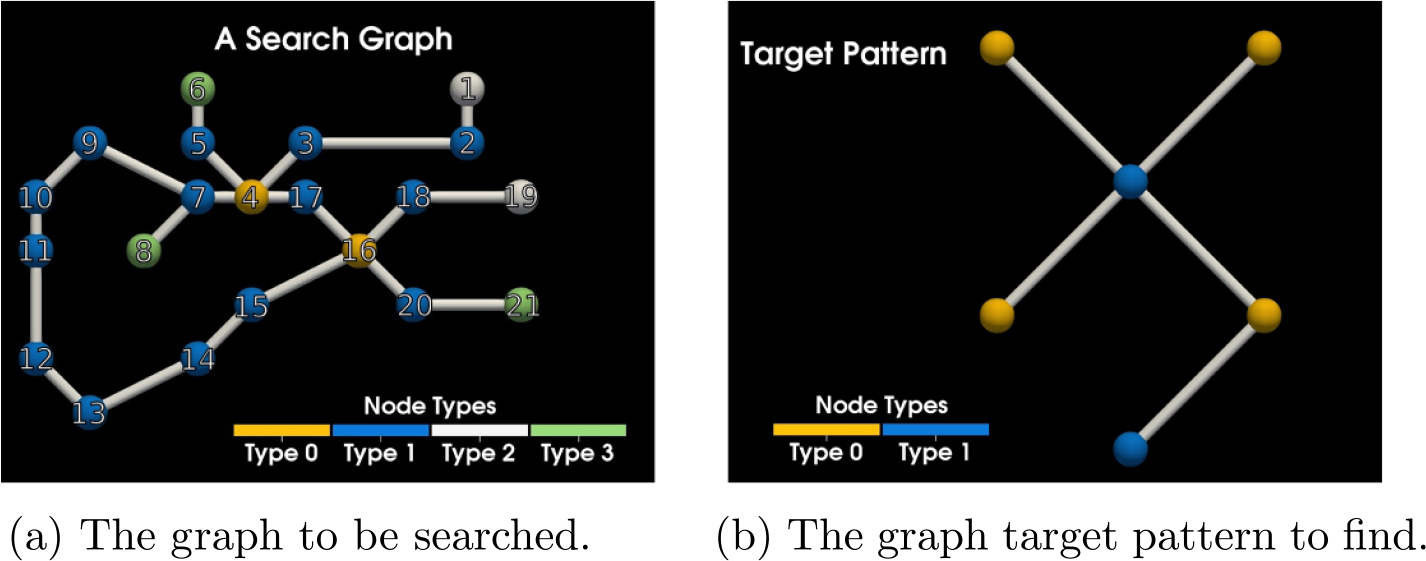
A side by side view of the search graph and the target graph.

We can demonstrate what we mean by finding the mappings f and g. In figure 4 we have a side by side view of the graph to be searched and an example of a target pattern. Figure 4(a) is $G_{SYS}$ and figure 4(b) is $G_{LHS}$ also known as the target graph/pattern.

**Figure 5.**
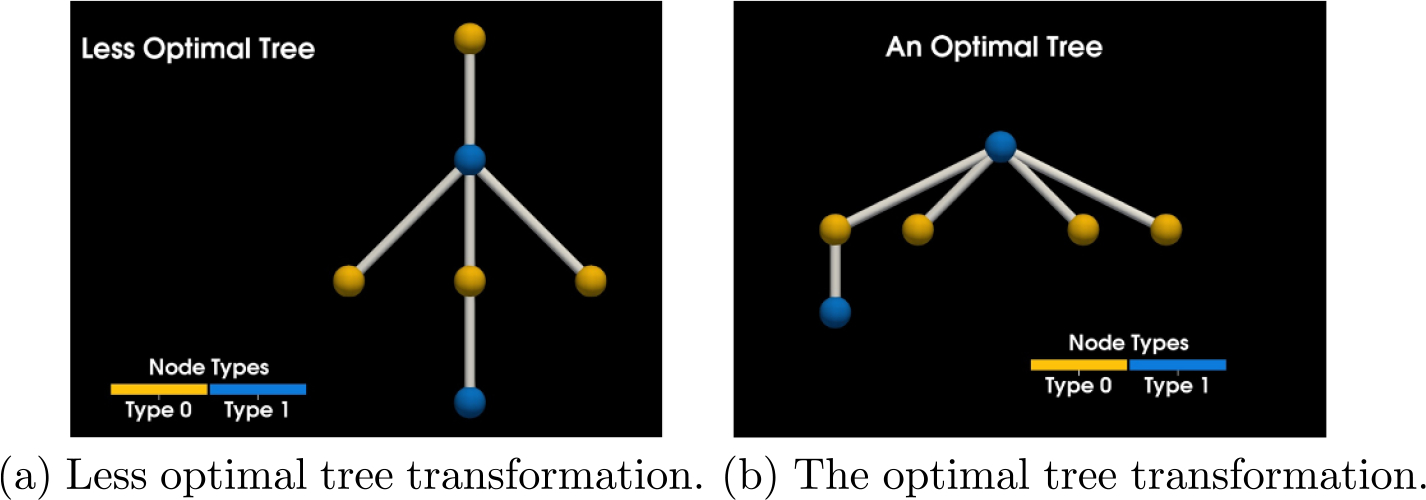
A side by side comparison of two spanning tree transformations.

Instead of trying to find the direct match for the target graph, we first find all matches g for some rooted spanning tree $G_{T}\subseteq G_{\text{LHS}}$, and then filter to ‘lift’ g to f if possible. We can see two examples of valid rooted spanning trees in figure 5. Figure 5(a) is an example of a less optimal root choice, whereas figure 5(b) is an example of an optimal choice for the tree. In general, the optimal choice is related to the height of the tree. The taller tree will be a more costly transformation for the search algorithm, since it will generate a more deeply nested search code.

**Figure 6.**
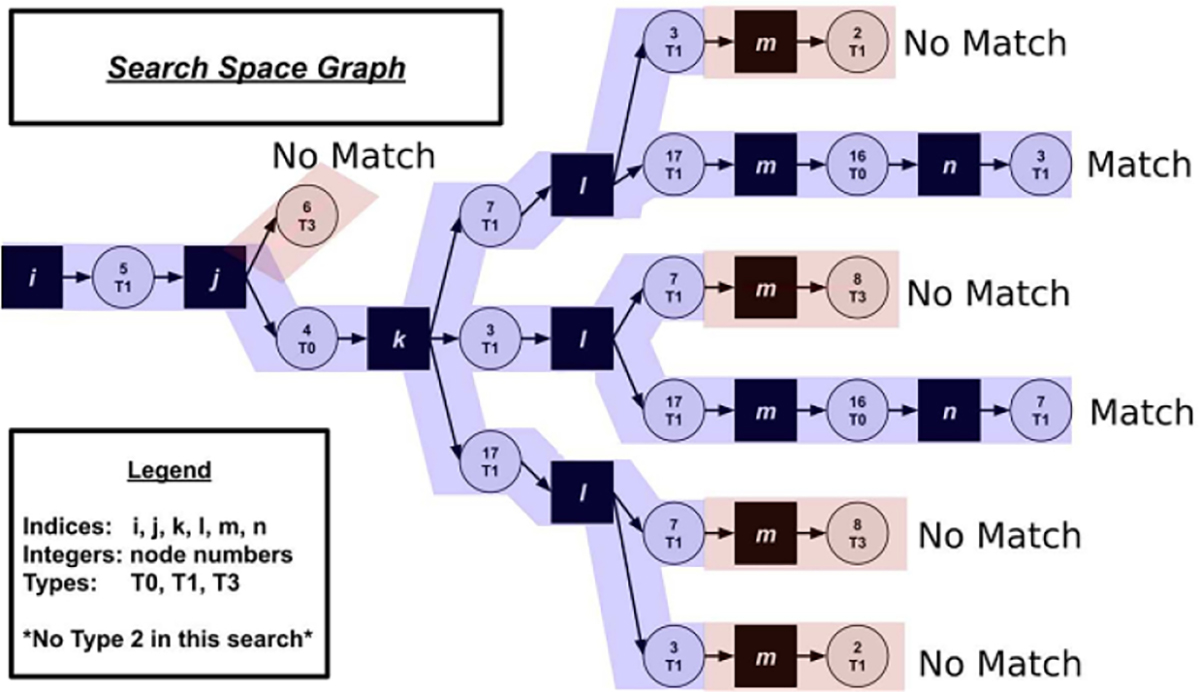
Example of how a search graph works to find an optimal path.

In figure 6 we can see what an algorithm searching the graph using the transformation of the target pattern seen in figure 5(a) actually does when we pick the starting node as depicted and start our rooted search. The rooted tree directly corresponds to our search path. The shorter the tree, the less deeply we need to search and the more search branches that can be pruned. We choose the less optimal tree to demonstrate this fact. Starting on node five in figure 4(a) we search and find two matches as can be seen in figure 6. A search started from each an every node in the graph would yield all possible matches. In this case, there are twelve matches (we include all valid permutations of a target match) and we finally have everything we need for a SSPR.

To solve the ODEs for deterministic grammar rules, we have included a state-of-the-art numerical solver. We use the suite of nonlinear and differential/algebraic equation solvers [35]. Solving functions are also manually written, but in future iterations a code generator would generate them directly from the DGG language.

In the absence of such a code generator, input is handled by configuration files. Simulation parameters are saved in a configuration file using JavaScript Object Notation (JSON) format. We use the library ‘simdjson’ [36] to read these files into our input file reader. Eventually, the JSON configuration files should be replaced by a more expressive language interface and a symbolic computer algebra system.

Our output file writer saves graphs in the visualization toolkit file format (VTU). VTU is a variation of an extensible markup language file. The VTU files are rendered using a combination of the visualization toolkit [37] and Paraview [38]. We save the ECC at the initial time step and save the system graphs periodically during simulations. Since the graphs are all spatially embedded, we can render a physical system as it evolves.

Additional metrics from the simulation such as the number of connected components (computed by YAGL), total node count, and counts of individual node types are collected during the run and transformed from C++ to Python Numpy arrays [39]. The arrays are used with Matplotlib [40] to visualize simulation results.

### 2.4. Steps for model creation

The DGG formalism defines how grammar rules are written and mapped into a master equation, but it does not define how to create a model. We identify six necessary steps for defining a model: (1) identify the initial conditions, (2) define a set of structure changing rules, (3) determine rate functions, (4) define the simulation domain’s geometry and topology, (5) set boundary conditions, and (6) determine time scale and other parameter settings.

The initial state can be generated by sampling from probability distributions or by other means, such as images. In our experiments we sample from probability distributions. The structure-changing rules should be defined using insight from biophysics. Rate functions determine how often stochastic graph rewrites occur and how parameters evolve deterministically. The rate functions are determined by theoretical or observed biophysical dynamics.

**Figure 7.**
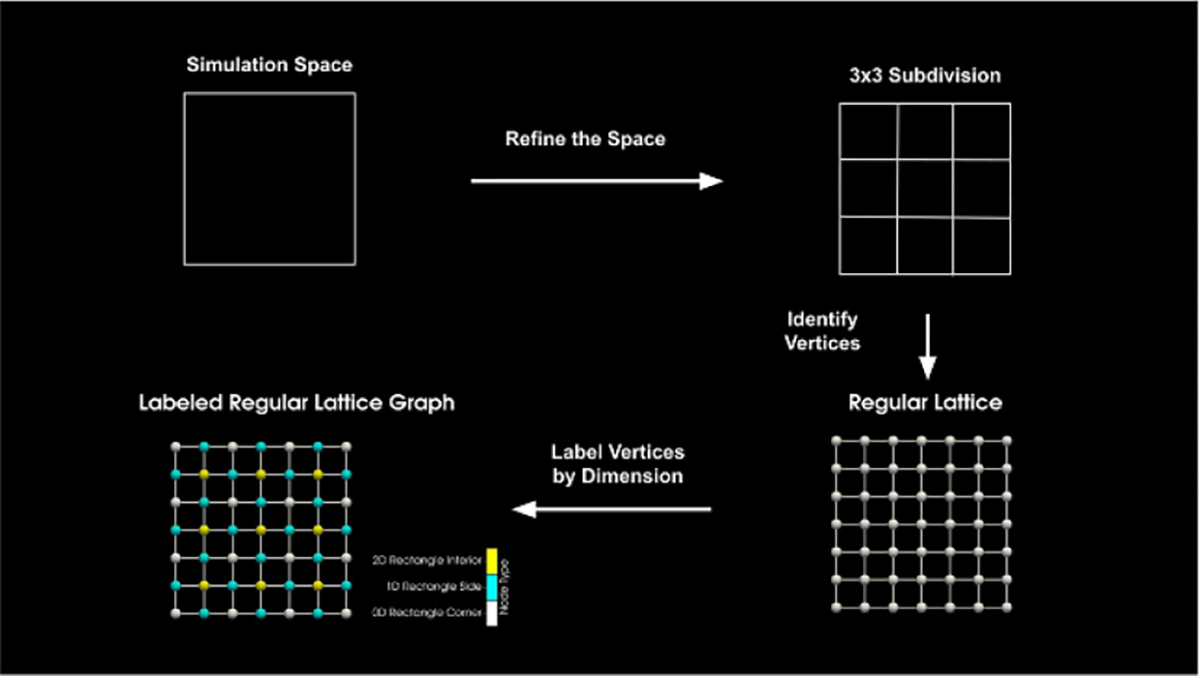
Plot of cell complex generation and labeling.

The geometry of the simulation domain represents the physical space to be simulated. We describe it using a labeled cell complex, which also encodes information about the simulation space’s connectedness and the dimensionality of each spatial domain. An overview of cell complex generation can be seen in figure 7.

The simulation algorithm has no restrictions on boundary conditions. Graph rewrite rules and domain constraints on the solving ODEs can be used to bound dynamics to a region in space. Periodic or reflective conditions are added in this way. In this work, rules have an implicit constraint on the ODEs: ODE solving only occurs within the non-ghosted geocells and any rule instance assigned to a ghosted geocell has a zero propensity. For more information on ghosted cells, see figure 3. Finally, in the current simulator, a JSON configuration file is used to set parameters such as time scale, rate function coefficients, and cell complex specifications.

### 2.5. Mapping biology and relevant physics to DGGs

In plant cells, the CMA plays an important role in cell division and determining shape [41]. An MT is a polymer composed of α and β tubulin proteins arranged in a cylinder of usually 13 longitudinal protofilaments [42]. MTs can be thought of as relatively stiff tubes around 25 nanometers in diameter. They can be represented in a graph as chains of stiff rod segments. Cortical microtubules (CMTs) in the CMA undergo structural dynamics such as treadmilling, zippering, induced catastrophe and crossover [6]—all of which can be represented as DGG rules.

The graph representation of CMTs is compatible with elastic dynamics and beam theory [43]. For example, in [28], MTs are represented as a string of points, using the standard formula for bending elasticity to allow MTs to bend under external forces and resist these forces elastically. The persistence length is one way to measure an MT’s resistance to bending, and it characterizes the length scale over which the MT maintains its direction while indicating its stiffness or flexibility. External forces can be caused by random thermal fluctuations, which can be described using the Boltzmann distribution. Thermal fluctuations can cause the MT to lose directionality over short length scales, resulting in a shorter persistence length [44]. The Boltzmann distribution plays a crucial role in determining the probability of the MT moving to a different energy state with a particular conformation and persistence length.

Currently, the model does not include the exact physics of these internal and external forces directly; however, these dynamics could be added as ODEs attached to nodes in the graph. The ODEs supported within the DGG formalism can also be extended to stochastic differential equations that include random fluctuations. In the current work, we have simply approximated fluctuations in the direction of the growing end by adding in small perturbations in the direction of growth when an instance of the stochastic rewrite rule in equation (10) is selected to occur.

While MTs are stiff (but still bendable) and can function to provide structure to a cell, they also have dynamic properties. In particular, it has been observed that MTs have the ability to undergo rapid growth and shrinkage, known as dynamic instability [45]. Dynamic instability and dynamic MTs provide a means for the cell to reorganize the cytoskeleton rapidly during cell division [42] or because of changes in the environment [46]. It has also been hypothesized that MTs can act as tension sensors [47], providing biomechanical feedback.

During dynamic instability, the MT is able to grow by rapidly polymerizing tubulin protein subunits bound to guanosine triphosphate (GTP) [42]. The cell must keep the concentration of GTP-tubulin high to allow for polymerization [42]. As long as the end remains stable, the MT will continue to grow, but as soon as instability is reached, the MT begins to splay apart and shrink [47]. In the grammar we encode these dynamics into our stochastic/deterministic growth/retraction rules. Alternatively, these processes are called rescues and catastrophes, respectively. Dynamic instability is regulated by MT associated proteins and incorporating them is a possible path for future work.

CMTs in the CMA also are subject to additional structural graph-changing dynamics. Three primary processes have been observed [6] and the mechanisms that control them are still a subject of debate. They are: zippering, crossover (junction formation), and induced catastrophe. If we let θ be the angle of collision and $\theta_{\text{crit}}$ be the critical angle of collision, zippering occurs at a higher probability with $\theta <\theta_{\text{crit}}$ and catastrophe and crossover occur at $\theta ⩾\theta_{\text{crit}}$ [48]. Grammar rules for the mentioned dynamics are provided in the supplementary material.

As mentioned in section 1, the CMA is associated with the cell’s plasma membrane. For our work, we restrict our simulation to be an idealized version of this region and leave an exact physical interpretation for future work. We focus on replicating the simplified dynamics mentioned and an use implicit capture condition for MT segments that reach the simulation boundary [49]. In the future, we could impose a more realistic boundary condition on the domain (such as capture and release) and add additional grammar rules to model transport dynamics within and between cells [41].

The following is an example of a stochastic dynamic graph grammar rule for growth:

**Positive MT Overgrowth**:

(10) $$\begin{array}{ll} & ({○}_{1}—{●}_{2})\;⟪\left(x_{1},u_{1}\right),\left(x_{2},u_{2}\right)⟫\\ & \;⟶({○}_{1}—{○}_{3}—{●}_{2})⟪\left(x_{1},u_{1}\right),\left(x_{2},u_{2}\right),\left(x_{3},u_{3}\right)⟫\\ & \;\text{with}\;\sigma \left(\frac{∥x_{2}-x_{1}∥}{L_{div}};k=10\right)\\ & \;\text{where}\;\left\{\begin{array}{c} x_{3}=x_{2}-\left(x_{2}-x_{1}\right)\gamma \\ u_{3}=\frac{x_{3}-x_{2}}{∥x_{3}-x_{2}∥} \end{array}\right. \end{array}$$

In equation (10), $\sigma (\cdot ;k)=1/\left(1+e^{-kx}\right)$ is a sigmoid activation rate function. Here $∥x_{2}-x_{1}∥$ is the length of the edge, $L_{div}$ is the maximal dividing length, and k=10 is a ‘gain’ parameter that determines how quickly the function turns on as the edge length gets close to the dividing length. Other k values could work, but we choose 10 for a rapid activation. The rate function increases rapidly when an MT segment grows too long, which increases the propensity that a growth rewrite rule-firing event will occur. The quicker the rate function activates, the sooner a new segment is added when the threshold is reached.

In the results section, we make use of the term ‘starting MT’. What we mean by ‘starting MT’ is a graph of the form:

(11) $$({◾}_{1}—{○}_{2}—{●}_{3})⟪\left(x_{1},u_{1}\right),\left(x_{2},u_{2}\right),\left(x_{3},u_{3}\right)⟫.$$

A ‘starting MT’ has a retraction node (closed square), intermediate node (open circle), and a growth node (closed circle). Edges simply represent relationships between nodes and the distance between the nodes in space can be computed by using the $l_{2}$ norm and node position vectors ${\vec{X}}_{i}$. As rewrite operations are applied to the MT using e.g. the rule in equation (10), growth is simulated.

**Figure 8.**
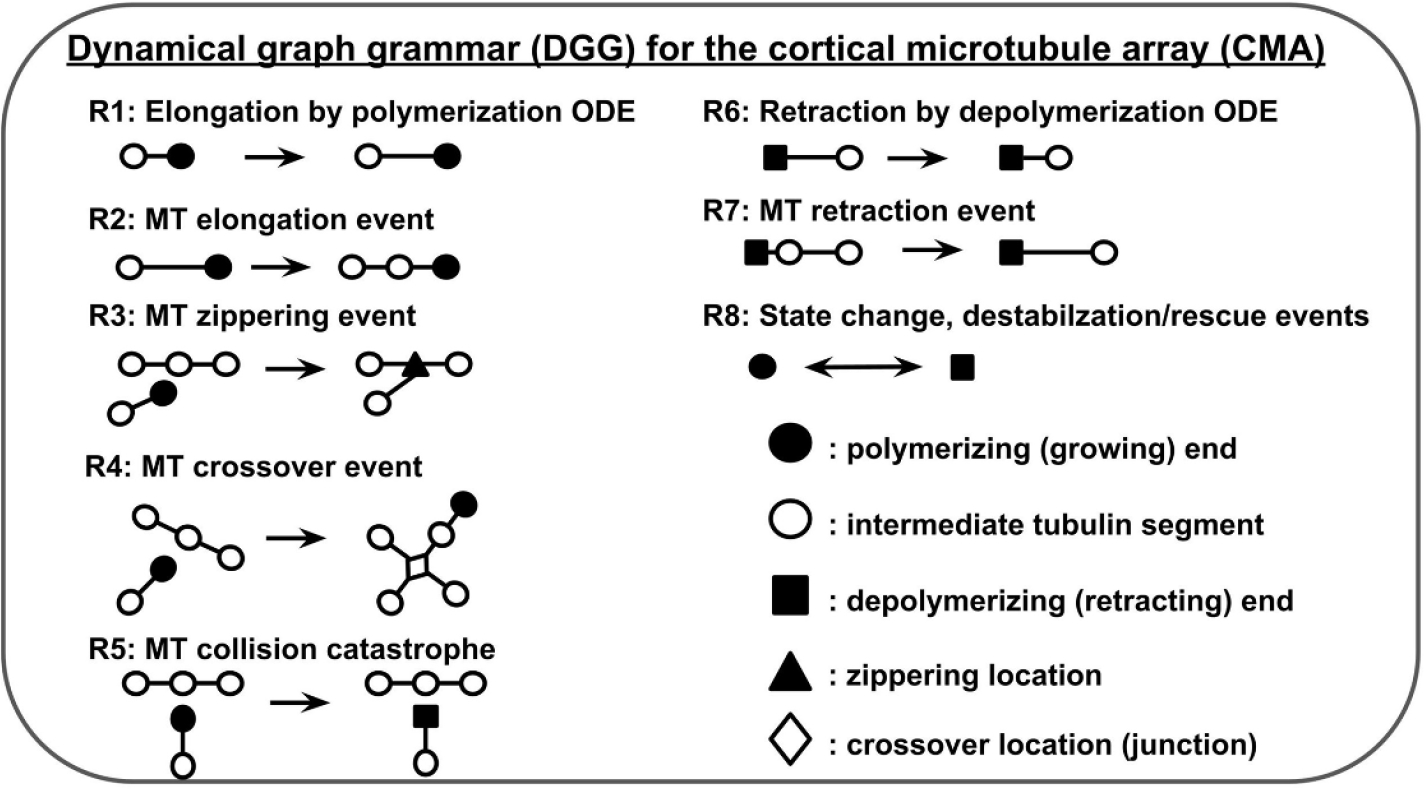
Summary of all the rules used in the CMA grammar.

In figure 8 we include a high level overview of all the graph rewrite rules used in the CMA grammar. Rule 1 is a deterministic rule that models the elongation of a polymerizing MT (growth) with an ODE. Rule 2 is a stochastic rule also used to model growth. When an MT segment becomes too long under the action of Rule 1, Rule 2 can insert another node and split the segment into two segments, as seen in equation (10). Rules 3 through 5 are stochastic rules that determine what outcomes may occur when a growing end of an MT comes close to two intermediate segments. In Rule 3, the outcome is zippering (bundling) if the MT comes in at a shallow angle. In Rule 4, the outcome is the MT crossing over the other and forming a junction. In Rule 5, the outcome is a catastrophe event and the colliding MT destabilizes and begins to retract. Rule 6 is another deterministic ODE-solving rule like Rule 1, but in this case it models retraction. Rule 7 is the stochastic version of retraction, like Rule 2, and determines what happens when an MT segment gets too short. Whereas Rule 2 adds a node, Rule 7 removes a node. Rule 8 is a reversible stochastic rule that allows the growing end and retracting ends to change states, to effectively model dynamic instability. Further details on ODEs, propensity functions, and rules may be found in the supplementary material.

## 3. Results and discussion

### 3.1. Overview

We have developed and implemented preliminary work on an approximate algorithm (Algorithm 2) implemented in C++ for accelerating the simulation of spatially embedded DGGs. Our simulator is also capable of running the exact algorithm (Algorithm 1), which is used as a baseline for the performance comparison. The current code is serial and single-threaded, leaving substantial room for parallel speedup due to the ‘parfor’ in algorithm 2. We have tested and evaluated our prototype simulator by running three experiments using the example CMA DGG found in the supplementary material. The CMA DGG uses artificial parameters to demonstrate proof of concept, and in the future more biologically inspired ones should be used.

In the first experiment, we simulated the CMA DGG several times for 1600 MTs and we evaluate the long-time behavior of the realizations. Our realizations include the change in the quantity of the five types of nodes of the microtuble graphs over time: retraction (negative growth), intermediate (interior nodes), elongation (positive growth), zipper (bundling), and junction (crossover). Numbers of each of the node types and the several realizations of the simulations for this experiment will be plotted in Section 3.2 below.

In the second experiment, we simulate the CMA DGG for 1600 MTs again, but with a low crossover rate and all other conditions remaining the same. We evaluate the long-time behavior of the simulation and compare it to the long-time behavior of the first experiment. A side by side comparison of the ending states will be shown in Section 3.3 below.

In the third experiment, we analyze the run-time performance of 3200 MTs with different domain decompositions. In Section 3.4 below, we show a quantitative comparison of performance using the exact algorithm (1 × 1 case) vs. the approximate algorithm (remaining cases) for the CMA DGG. The approximate algorithm allows for speedup by breaking the system into well-separated reaction sub-systems, which obviates the need to evaluate most possible matches, and by firing some rules out of order as defined by operator splitting, at the cost of accuracy.

### 3.2. Experiment 1: long-time network formation

**Figure 9.**
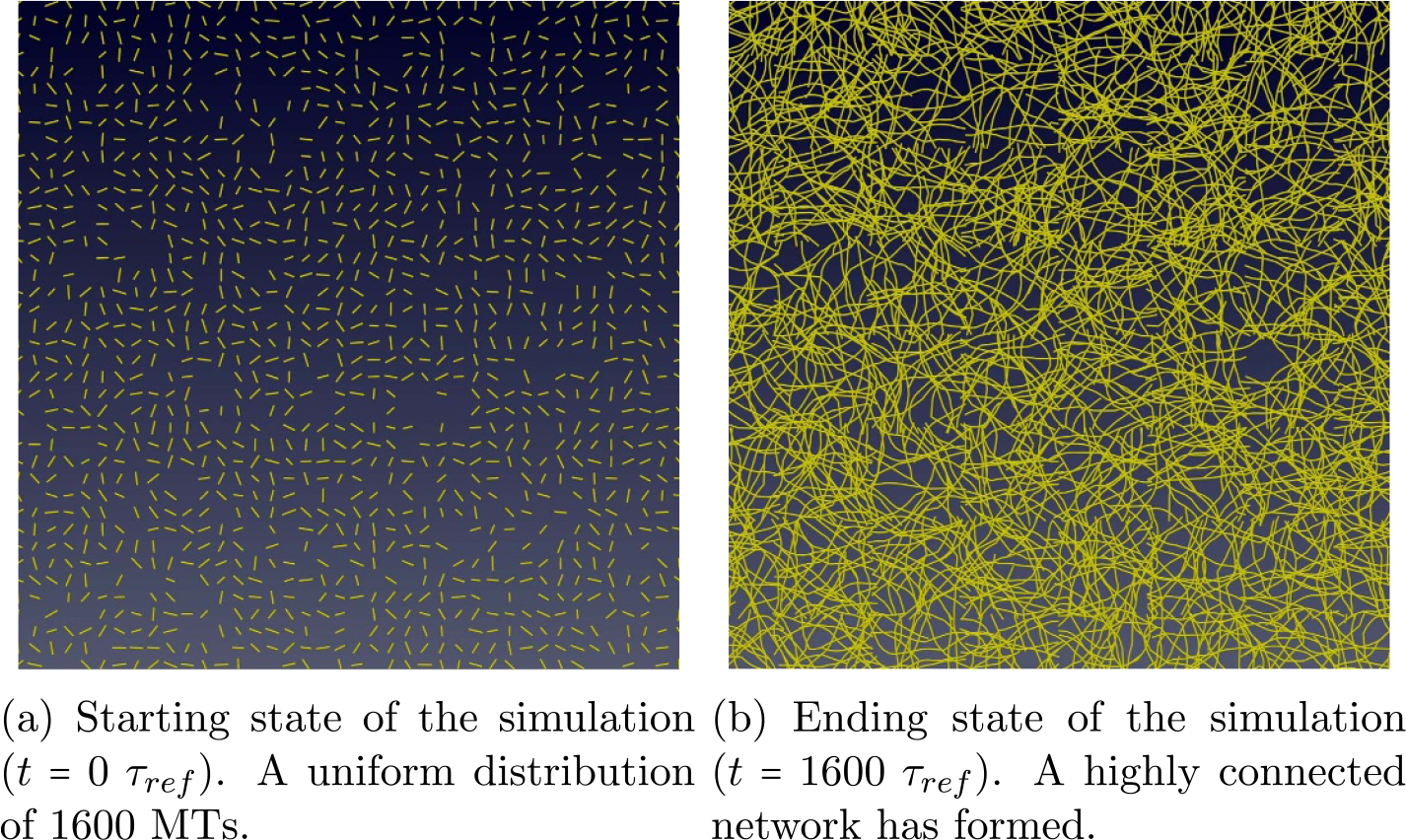
Side by side comparison of beginning and end state of the CMA DGG simulation of 1600 MTs for realization 3.

We initialize each simulation of the system with 1600 MTs. An example of the starting state of a realization is seen in figure 9(a). The initialization follows a uniformly random distribution over the domain space. The domain is Ω={0⩽x,y⩽100∣x,y∈R}, a 100 × 100 square area in $R^{2}$. For each simulation, we subdivide the domain into a uniform 8×8 grid. Let $\Omega_{i}$ be the ith 2D simulation cell, with dimensions 12.5 × 12.5. Further, |Ω|=64 and ${⋃}_{i=1}^{64}\;\Omega_{i}=\Omega$. The subdivided domain is then transformed into a cell complex and expanded.

The average MT connected component density per highest dimensional cell is $\frac{25\;MT}{\left|\Omega_{i}\right|}$. The average node density is then $\frac{75\text{Nodes}}{\left|\Omega_{i}\right|}$. The initial average MT density is chosen to be $\frac{25\;MT}{\left|\Omega_{i}\right|}$, rather than a larger quantity to keep the starting MTs well-separated and allow for polymerization to occur before junction/zipper formation begins (room for growth). We take the boundary conditions to be the capturing condition [49].

#### 3.2.1. System dynamics and long term behavior

For the experiment, we let the simulations run for 1600 units of simulated time. We define one unit of coarse scale simulated time $\tau_{\text{ref}}=vl$ to be the time it takes one MT positive node to move a segment distance l, given a velocity v. Conceptually, this is similar to the Courant–Friedrichs–Lewy condition in numerical PDEs [50]. Under our constraint, we ensure that not too much happens in the system in one time unit, as required by our approximate simulation algorithm.

Propensity function model parameters are chosen to evaluate the simulation algorithm and code, by equally exercising all the DGG rules derived from recent literature, rather than to represent biophysical knowledge.

**Figure 10.**
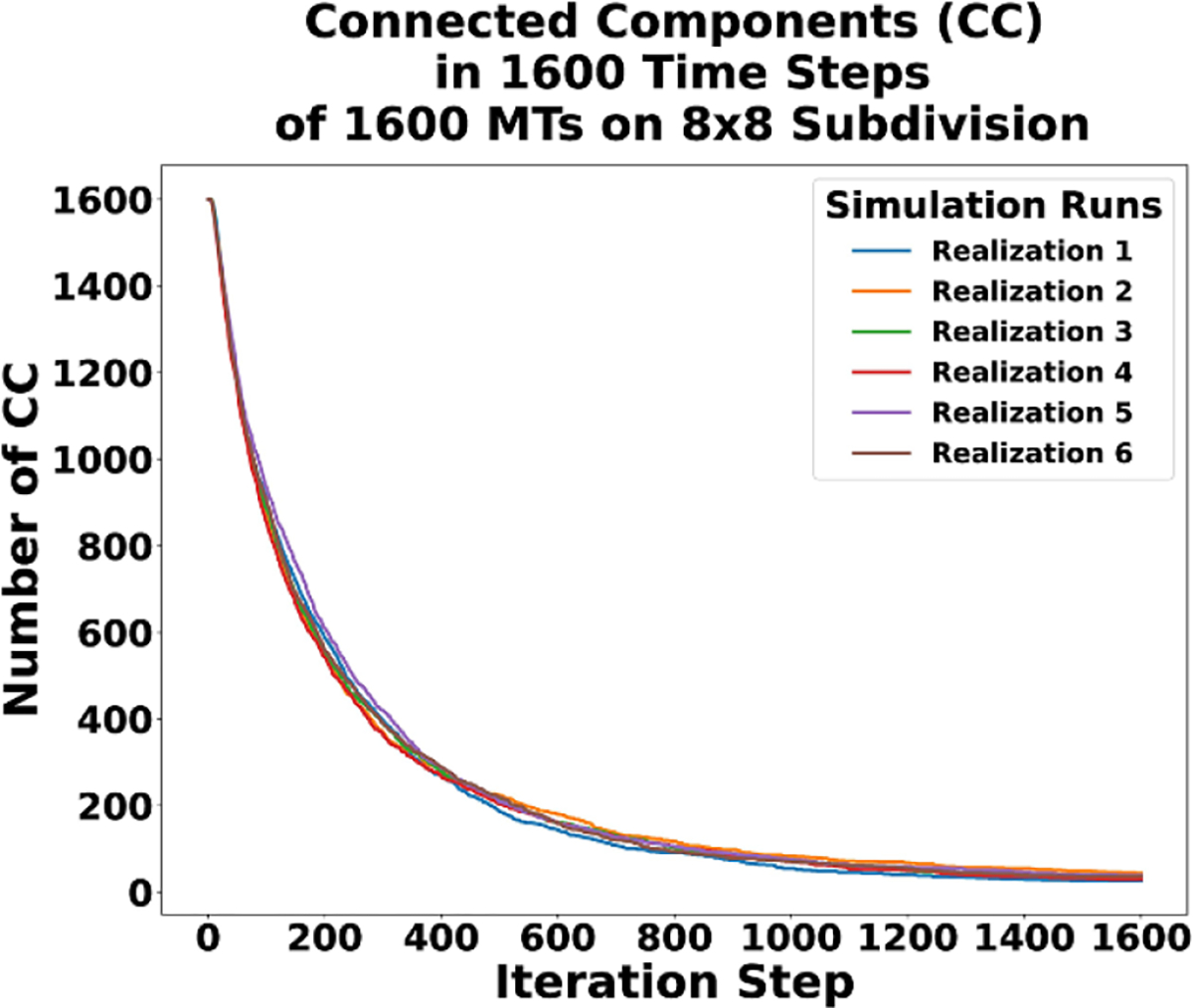
Six realizations of the change in connected components over time.

Figure 9(b) is the final state of the realization of the third simulation after $time=1600\tau_{\text{ref}}$. It shows network formation and the onset of a steady state in the long term behavior of the system. A side by side comparison of the starting state and ending state can be seen in figure 9. The starting state in figure 9(a) shows 1600 disconnected starting MTs uniformly distributed. In figure 9(b) the ending state consists of a highly connected network, and the same behavior occurs in the other realizations. We verify this with a plot of the connected components for all simulations in figure 10.

**Figure 11.**
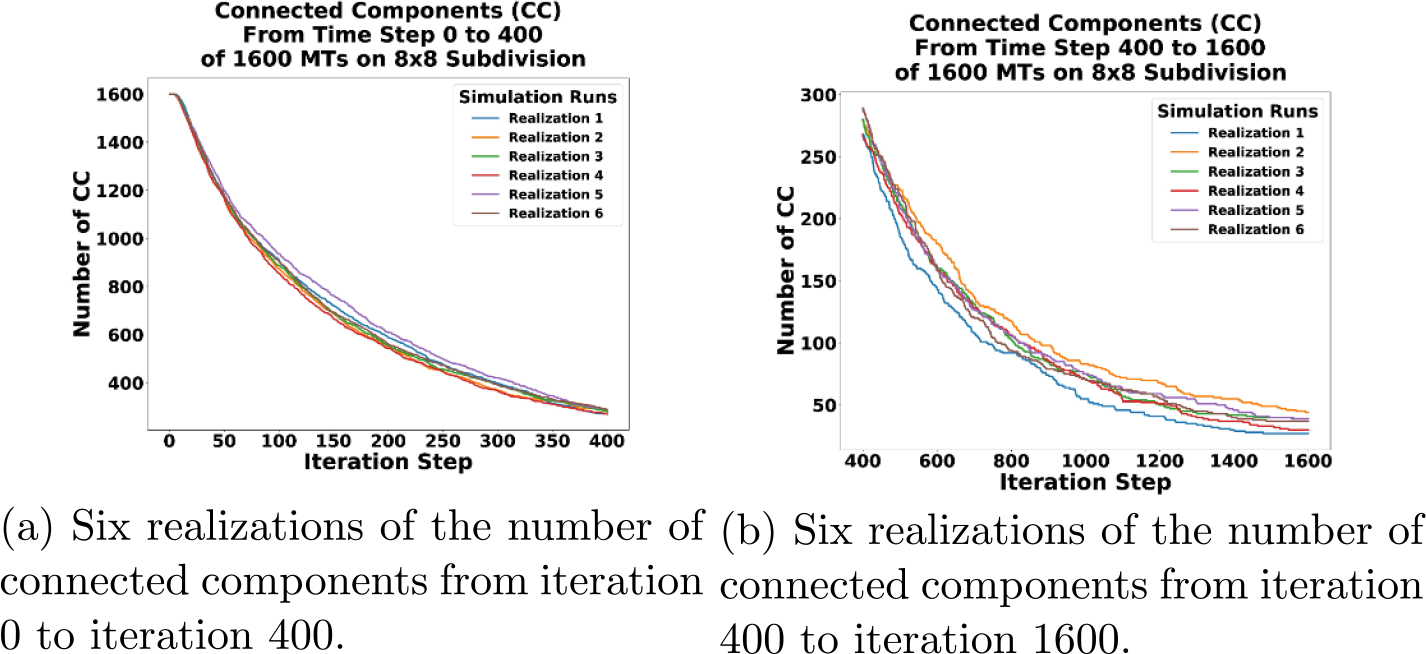
Zoomed in plots of the beginning and end of six realizations of the number of connected components changing over simulation iterations, where one realization becomes a fully connected network.

In figure 10, we start with 1600 connected components for each simulation. One connected component for each MT as exemplified in figure 9(a). Over time, we see the connected components decrease and trend toward the long-time behavior of a highly connected network. A fully connected network is expected to emerge if we ran the simulations for longer. To make the difference in connected components of each realization clear, figure 11 is included. In figure 11(a) all realizations are plotted from the beginning to iteration 400. Figure 11(b) plots the realizations from iteration 400 to the end and distinctly indicates the slight difference between realizations in number of connected components over time.

**Figure 12.**
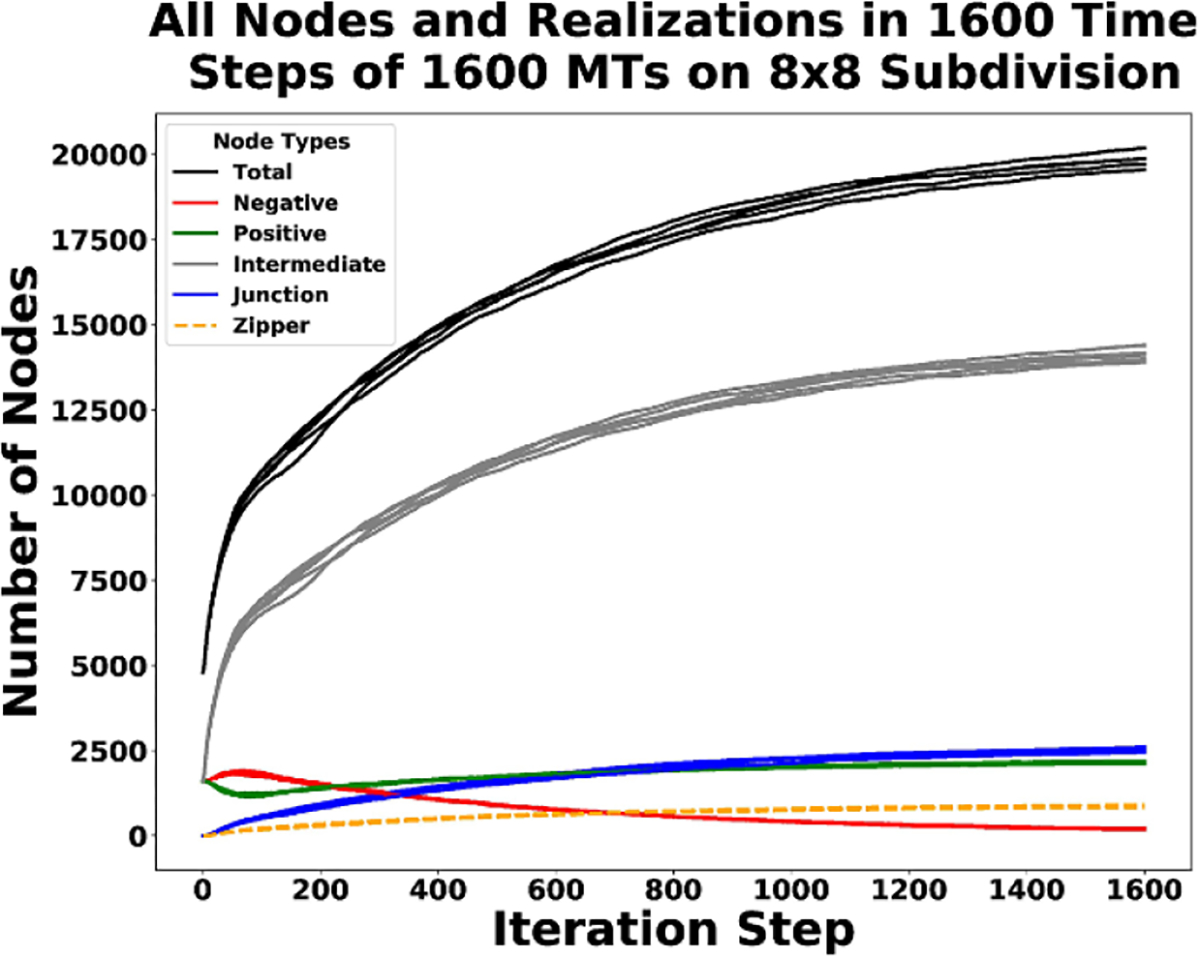
Plot of long term behavior of all node types, including all six realizations of the CMA DGG simulations.

In figure 12 we see the long term behavior of each node type in the system for all of the realizations. The plot shows how many of each node type we have in the system after every iteration. The top line in the plot is the total number of nodes. In each simulation, we start with 4800 nodes (three for each starting MT—equation (11)). In all of the end states of the realizations we have over 17 000 nodes, indicating an average increase by at least a factor of three.

**Figure 13.**
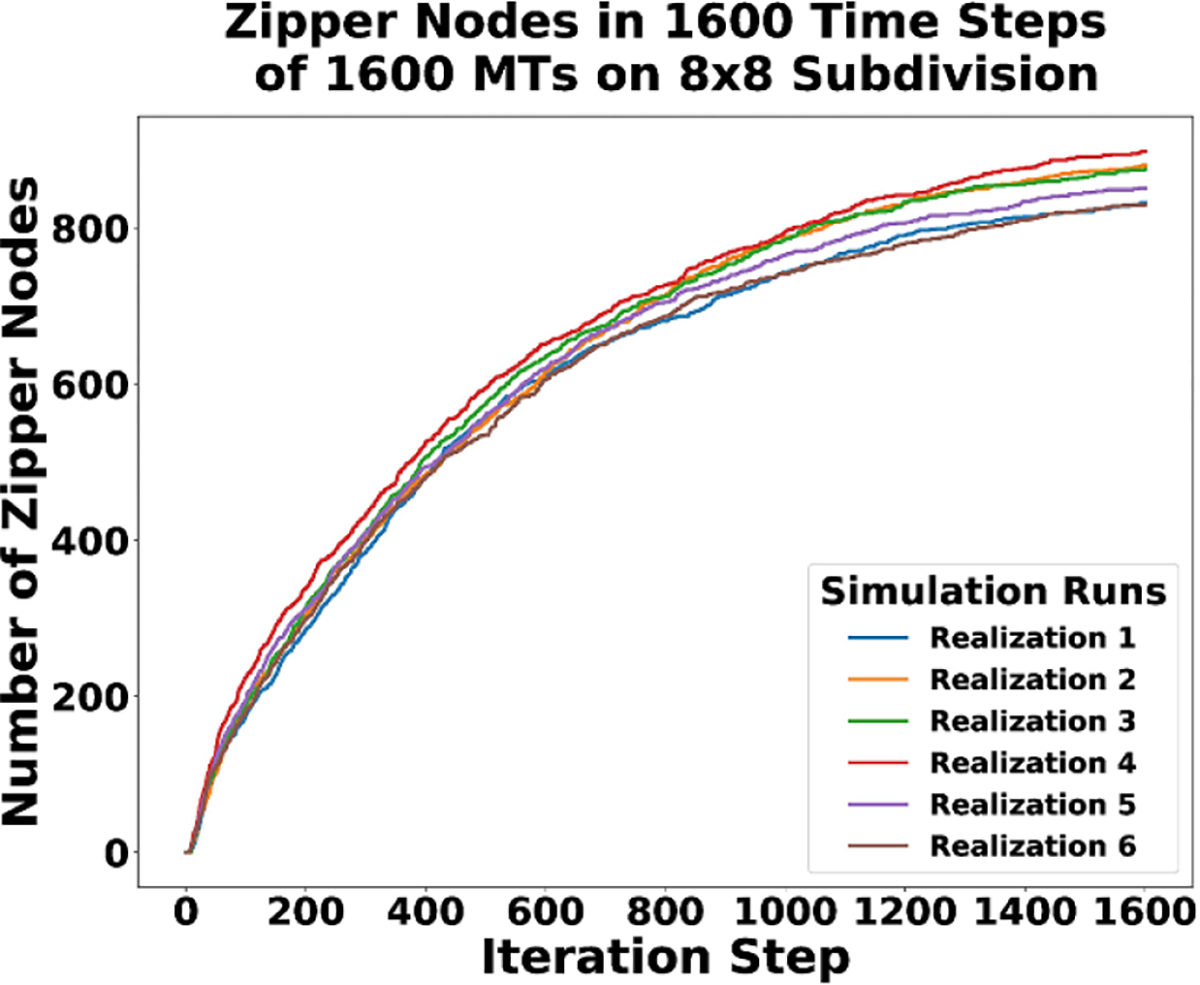
Plot of the change of zippering nodes over time for all six realizations of the CMA DGG simulations.

The number of junction nodes in the system is different than the number of zippering nodes (on average three times as many junction nodes as zippering nodes), but they still follow a similar long-time trajectory as seen in figure 12. Since the zipper node dynamics are similar to the junctions, we only provide analysis for one. In figure 13, we can see how the zipper nodes change for each realization. Figure 13 indicates the number of zippering nodes increase rapidly at first and then begins to slow as we reach the long term asymptotic behavior.

**Figure 14.**
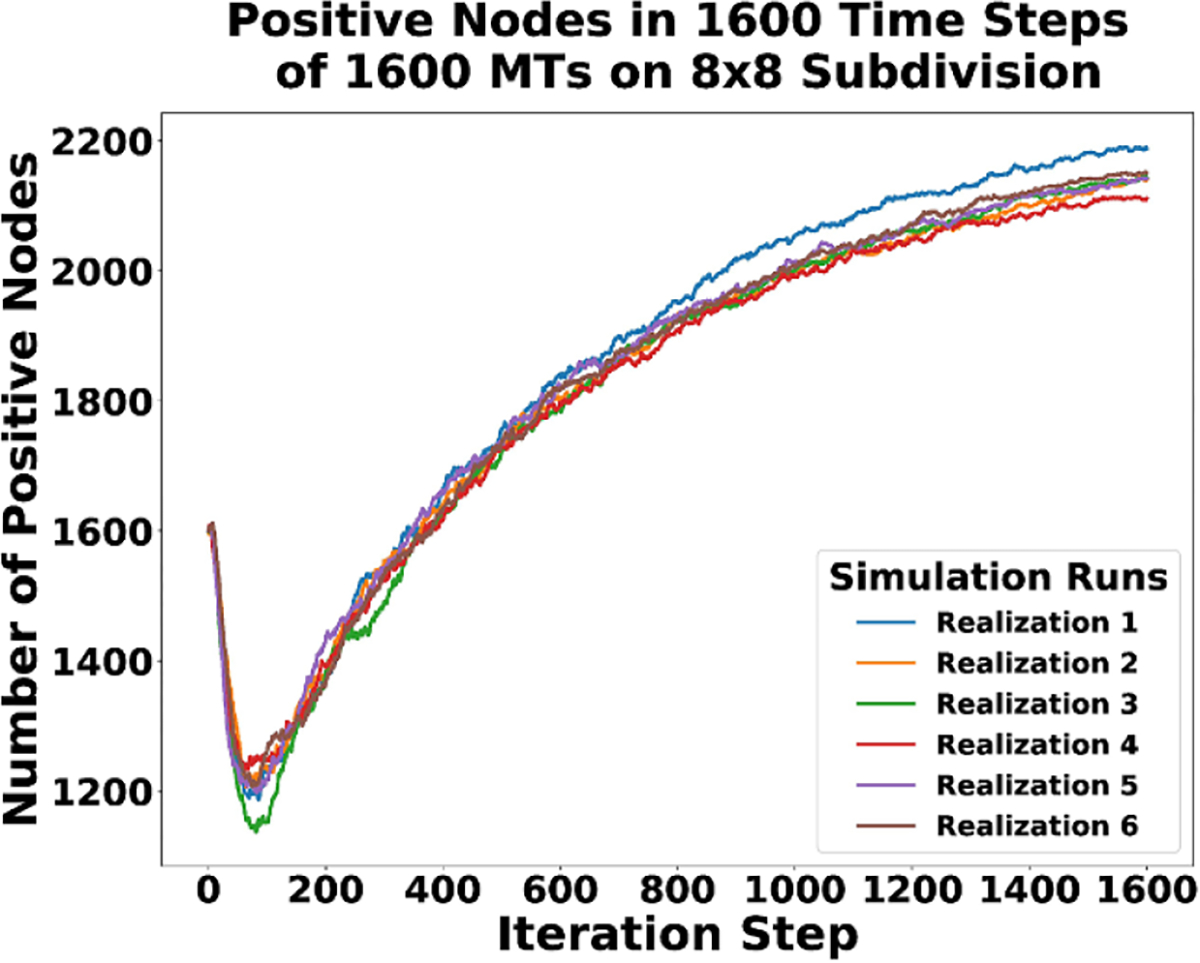
Plot of the change of positive nodes over time for all six realizations of the CMA DGG simulations.

Figure 14 shows how the number of positive growth nodes in the system changes. The positive nodes are primarily responsible for the creation of new MT segments because of their participation in the growth rule, with the only other creation of new segments occurring during a junction/zippering rule firing. The of rate of MT polymerization was set to be four times as fast as the rate depolymerization. If the capturing boundary condition (BC) had not been imposed, the number of positive nodes in the system may have grown without bounds, since the rate of polymerization exceeds that of depolymerization. There is also a state change rule, which occasionally switches a negative end to a positive end or a positive end to a negative end.

Initially, we see a drop in the number of positive nodes at the beginning of the simulation. The cause is likely a combination of the state change rule, along with the capturing BC. Eventually the growth recovers, and over time the positive nodes begin to again be captured by the BC or restricted in their directional dynamics due to the onset of zippering and junction formation. Any time a junction or zipper is formed, it creates a permanent and on average irreversible directional barrier for the growing end. The barrier is on average irreversible, since there is no CMA DGG rule yet included to reverse the formation of a junction or zippering node. The growing rule does include a stochastic unit vector wobble, which means an MT could eventually circle around to form a new junction or zippering node, but that behavior is not likely. Thus, in general, the number of new positive nodes added into the system is expected to decrease over time and the total number of positive nodes is expected to reach a steady state depending on the particular realization, as seen in figure 14.

**Figure 15.**
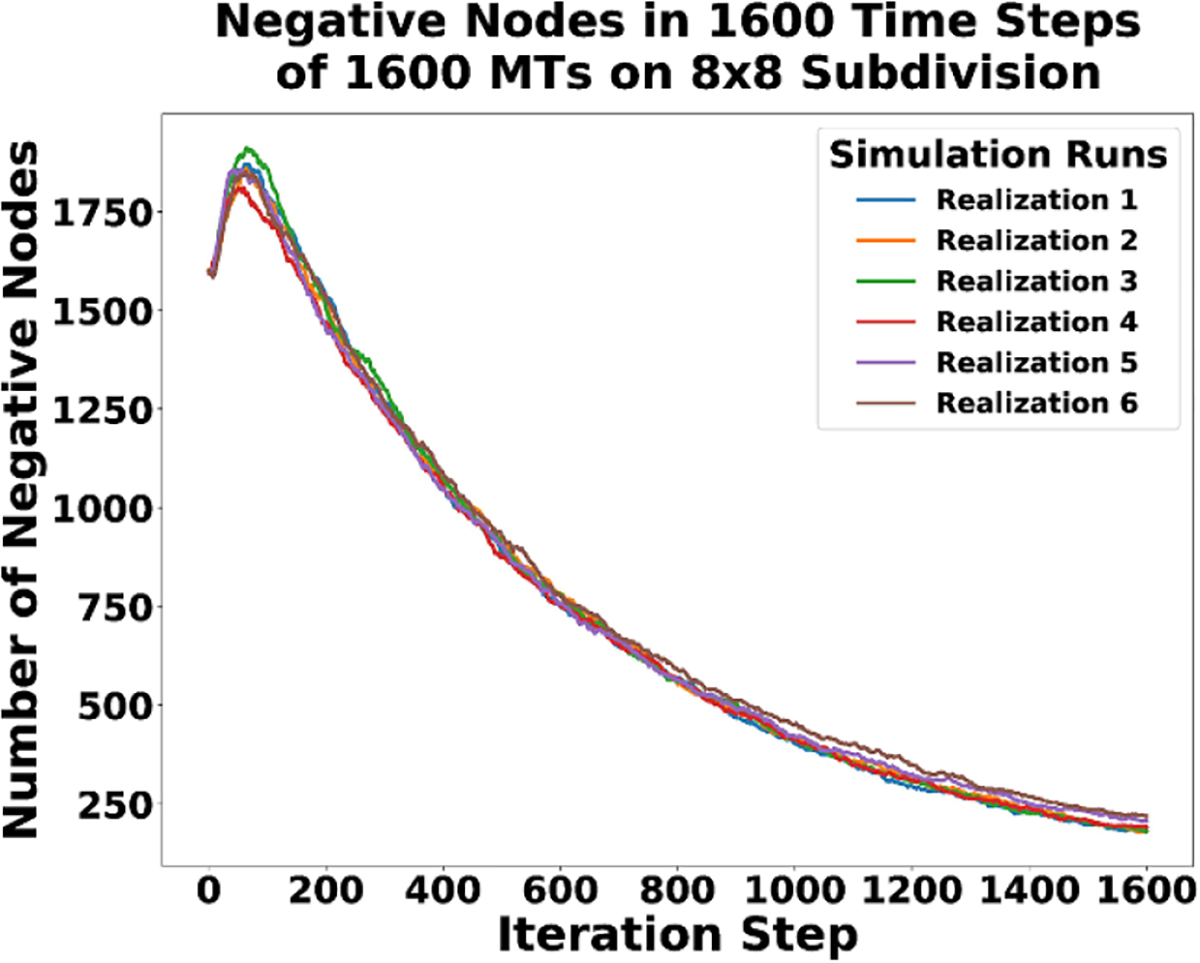
Plot of the change of negative nodes over time for all six realizations of the CMA DGG simulations.

The negative nodes in the simulations follow dynamics similar to positive nodes, but delayed in time (figure 15). The time delay is likely caused by the slower rate of retraction as compared to growth. Each simulation starts with a fixed number of negative nodes that should decrease over time due to the BC and junction/zipper formation. We see this exact behavior, but with a slight initial increase in negative nodes before long term decay into a steady state. If the simulations ran longer, it is expected that no negative nodes would exist, because they state-changed to positive and got captured.

**Figure 16.**
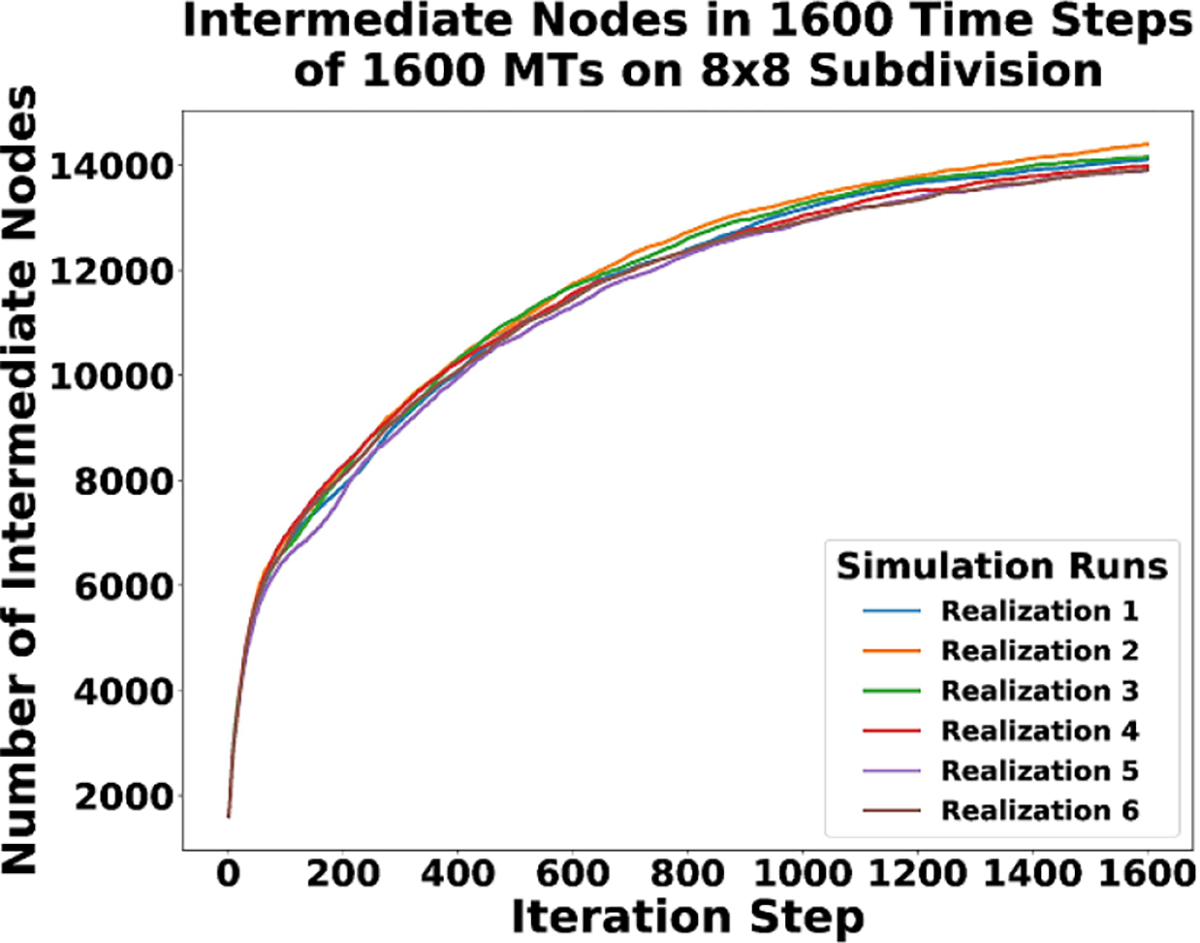
Plot of the change of intermediate nodes over time for all six realizations of the CMA DGG simulations.

Finally, figure 16 is a plot of how the number of intermediate nodes change over time in each realization. In the CMA DGG simulations presented, the number of intermediate nodes in the system directly corresponds to the number of MT segments that exist. The growth rule functions to add more intermediate nodes; however, the zippering/junction rules and the capturing BC lock the system into place and slow growth. Consequently, network formation is encouraged, but longer term growth is discouraged. So the more a network begins to form, the more intermediate nodes we get. As it forms, the addition of intermediate nodes decreases. Eventually a steady state is reached and the number of intermediate nodes existing stabilizes.

#### 3.2.2. Reactivity and iteration analysis

The MT dynamics of even a relatively simple system can be complicated. More complex dynamics require more computation and make performance a concern. We measure performance over the duration of one simulation step of $\tau_{\text{ref}}$ time, an *iteration*. We use reactivity per iteration as a quantitative measure of performance, where *reactivity* is the wall clock time of an iteration. Wall clock time is an appropriate measure because iteration time is correlated to the number of reactions occurring. For example, preliminary experiments with a grammar including a katanin-mediated severing rule had reactivity increase rapidly.

**Figure 17.**
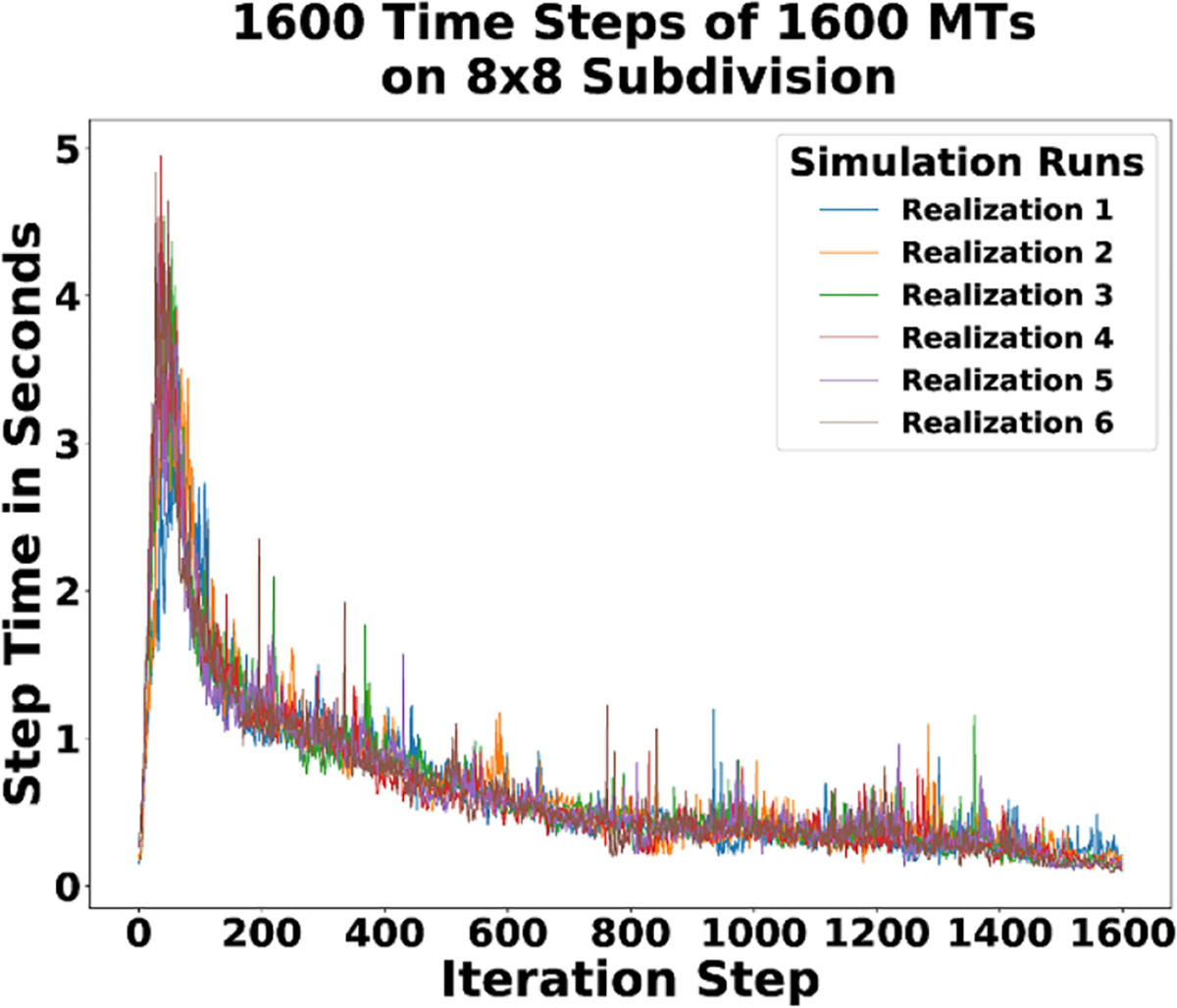
Plot of run-time per simulation iteration of six realizations of the CMA DGG simulations.

For the previous experiment of 1600 MT, the initial density was chosen to be low enough for each simulation to keep MTs in the starting state far apart from interacting with each other and to leave room for growth. Figure 17 shows how the system run-time dynamics change over time for different realizations. The plot is the actual real world run-time per iteration. The reactivity plotted is the sum of the run-time for all of the geocells in an iteration. In general, the exact algorithm run within a geocell of a given operator-split dimension for any subdivision may not run in the exact amount of time as other geocells in that same dimension; however, in this experiment they should on average because the MTs are initially uniformly distributed (figure 9(a)).

In figure 17, the reactivity of the system increases rapidly over most of the first 50 iterations. The reactivity observed is reflective of the dynamics as detailed in figure 12 and a consequence of MTs growing at a rate faster than they shrink. The peak reactivity occurs just before iteration 50. After the peak, the network begins to form as irreversible junction/zippering nodes are created and the reactivity of the system decreases. By around iteration 100 and onwards, the reactivity trends downward towards a steady-state, which corresponds to the realized system dynamics in figure 12 and the network in figure 9(b).

### 3.3. Experiment 2: long-time local alignment

We use the same parameters and CMA DGG (figure 8) as experiment 1, but with the rate of crossover events lowered to near zero. Effectively, zippering and catastrophe events are favored. The starting state is the same as in figure 9(a).

**Figure 18.**
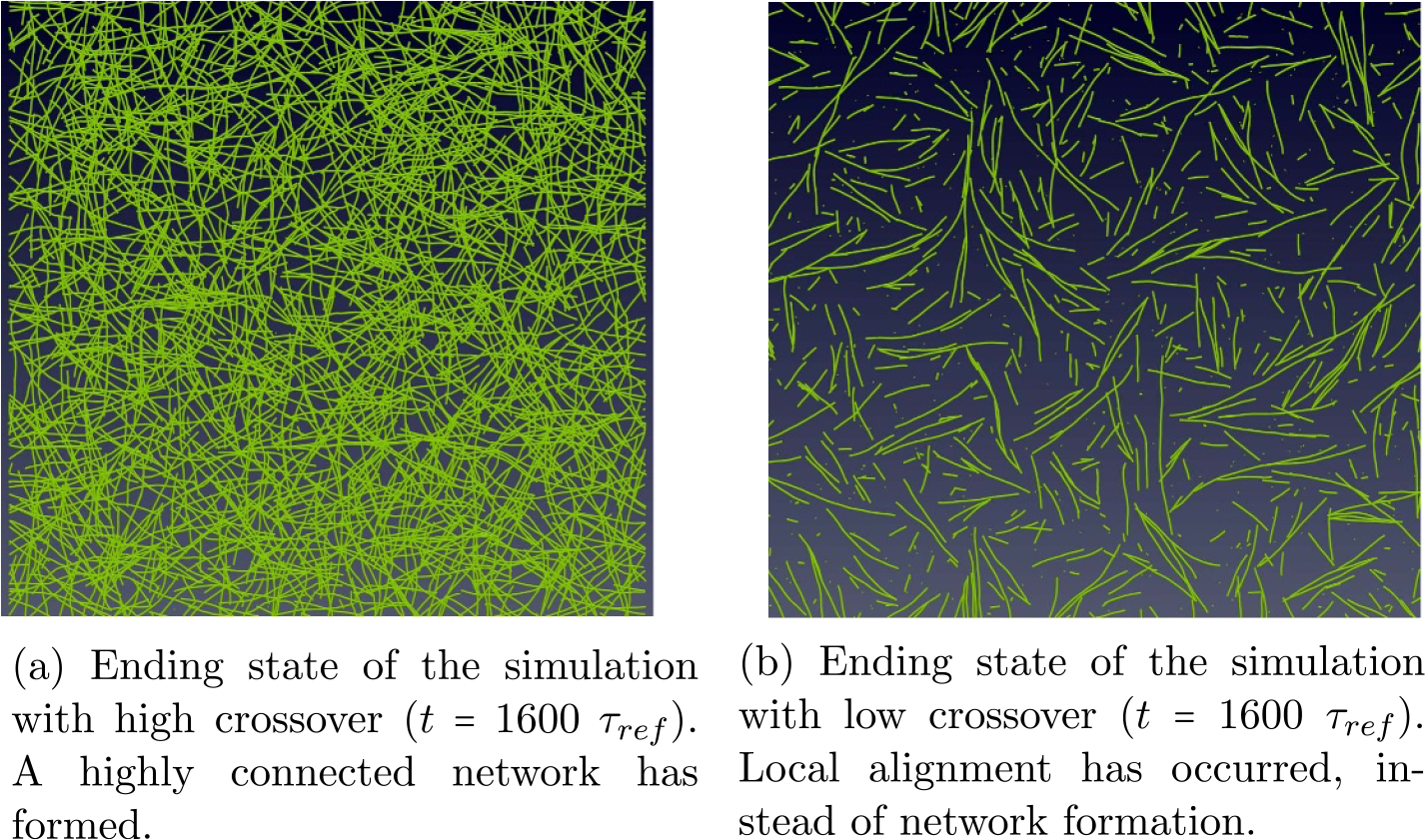
Side by side comparison of the end states of the CMA DGG simulation of 1600 MTs on a 100 × 100 unit grid showing the effect crossover has on the system.

Figure 18 compares the ending state of the system with a low crossover rate to the system with the original crossover rate. In figure 18(a) a highly connected network has formed, whereas in figure 9(b) we can see that lowering the crossover rate leads to less connectivity, inhibits network formation, and reduces the number of surviving MTs. Significantly, figure 9(b) exhibits localized alignment where the first experiment did not.

We compare how aligned the two ending states are by computing an MT orientation correlation function defined as the squared cosine between the orientation of the MT segments, and average within bins of roughly constant distance. (This measure can be derived as the trace of the product of the two rank-one projection matrices defined by the two unit vectors; it is invariant to sign reversals of these unit vectors.) The function measures on average how ‘aligned’ MT segments a distance away are from one another. The square is needed to remove anti-symmetry, since nearby MTs may be aligned but in anti-parallel directions, and anti-parallel alignment is not visibly distinguishable from parallel alignment in typical MT imagery. Values close to 0 using this measure indicates orthogonality and therefore no alignment, whereas values close to 1 indicate complete parallel or anti-parallel alignment. Typical intermediate values for lines at 45° (equivalently 135°) to one another are 1/2. Consequently, we subtract 1/2, the ‘uncorrelated’ value, before averaging within distance bins of width defined by the reaction radius, and fitting an exponential decay as a function of distance.

**Figure 19.**
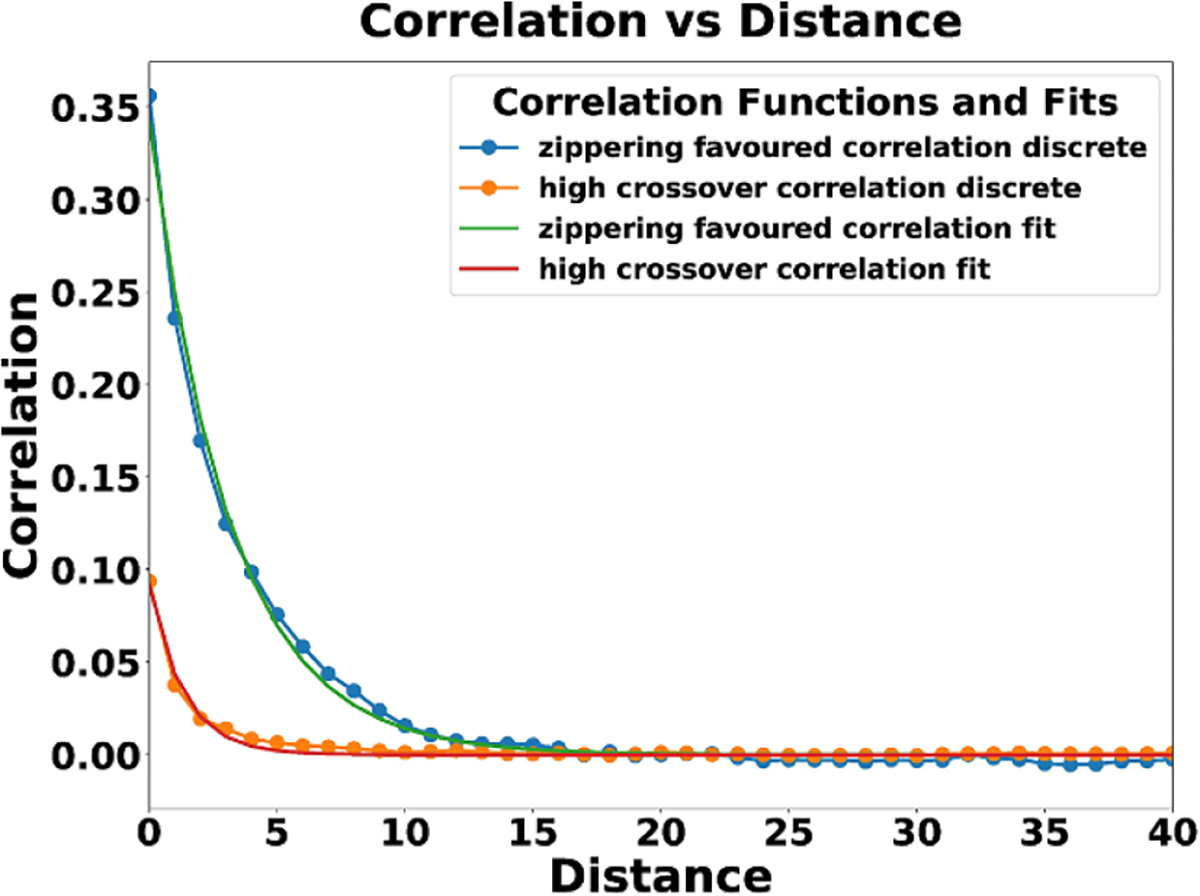
Sampled correlations of alignment over distance and their exponential fits of ending system states seen in figure 18.

We can see the fitted correlation vs. distance functions in figure 19. When we fit to an exponential decay on figure 9(b), we get the fit $c(d)=0.34e^{\left(-d/3.14\right)}$ with a mean absolute squared error (MASE) of 0.797. We initially have high correlation and then a quick drop off with a correlation length $\xi_{2}\approx 3.14$ with a standard error (SE) of 0.06 for the plot in figure 19. When we fit to an exponential decay on figure 18(a), we get the fit $c(d)=0.09e^{\left(-d/1.34\right)}$ and an MASE of 0.709. There is a much lower initial correlation and then a rapid drop off with a correlation length $\xi_{1}\approx 1.34$ with an SE of 0.039 for the plot in figure 19. Even if we were to very conservatively zoom in on figure 18(a) by a factor of 1.6 to equalize the number of MT segments in each window, the resulting correlation length of ${\overset{ˆ}{\xi }}_{1}\approx 2.14$ is (as detailed in the Supplemental Material) many standard deviations short of $\xi_{2}\approx 3.14$; even more so for $\xi_{1}$ vs. $\xi_{2}$. Similar statistics, among many others (e.g. [51] for graph structure), could in the future be used to compare model-generated with biological-experiment imagery.

Our comparison indicates that zippering and catastrophe may lead to local alignment, partially supporting the ‘survival of the aligned hypothesis’ [7]. The results seen in figure 9(b) also look closer to what a real system of CMTs might look like. Alternatively, experiment 1 indicates that zippering, crossover, and catastrophe lead to network formation. The addition of a selective katanin mediated severing rule using an alternative hypothesis [8] also has potential for global alignment of MTs in the system after the network has formed, but is a topic for future work.

### 3.4. Experiment 3: approximate vs. exact performance

As a computational performance experiment, we started the simulation with an initial condition of 3200 MTs random uniformly distributed across a 100 × 100 unit grid. We use the same grammar rules and parameters as the first experiment, along with identical node-capturing boundary conditions.

**Figure 20.**
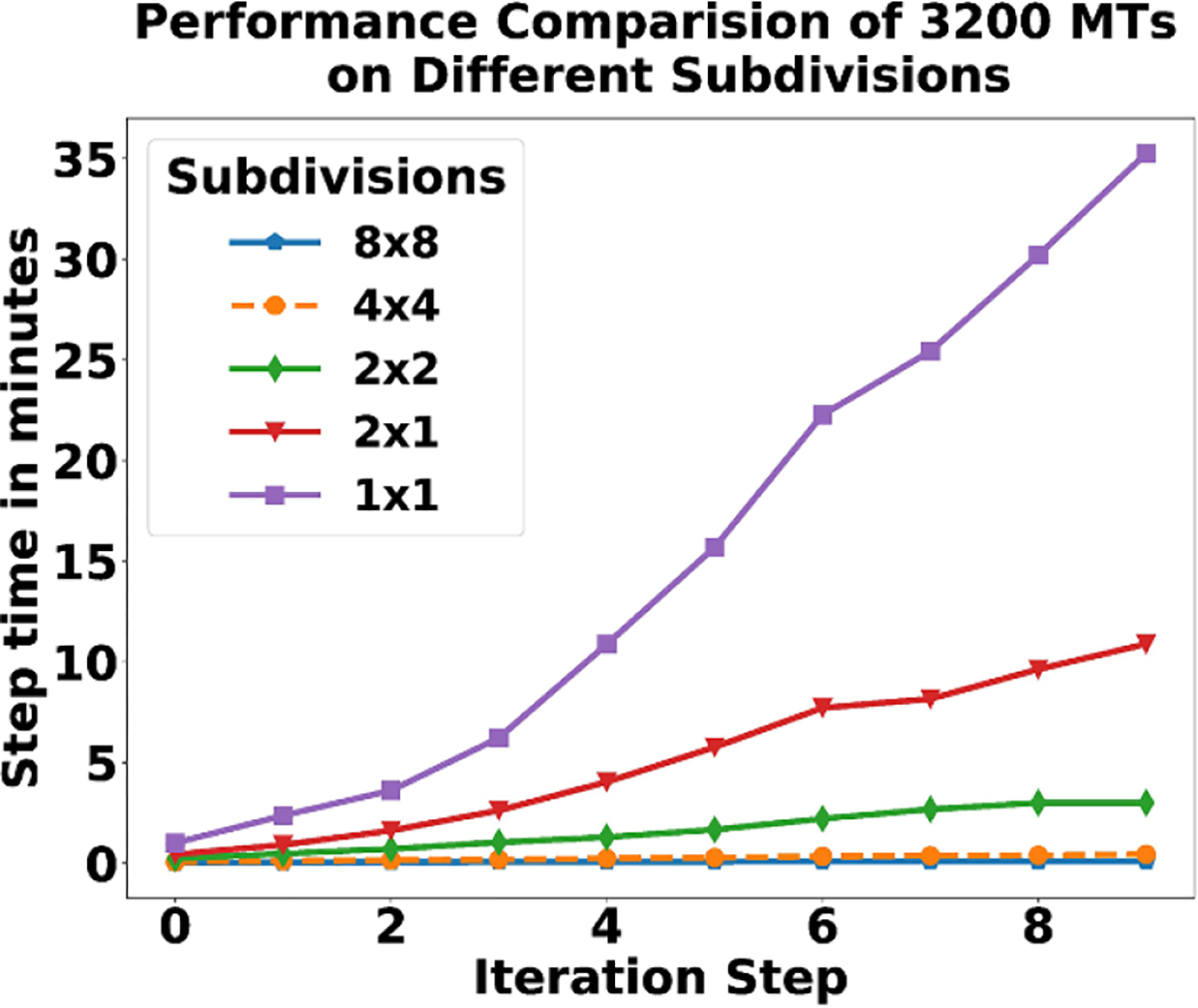
Plot of performance analysis of five separate simulation runs for 10 iterations.

**Figure 21.**
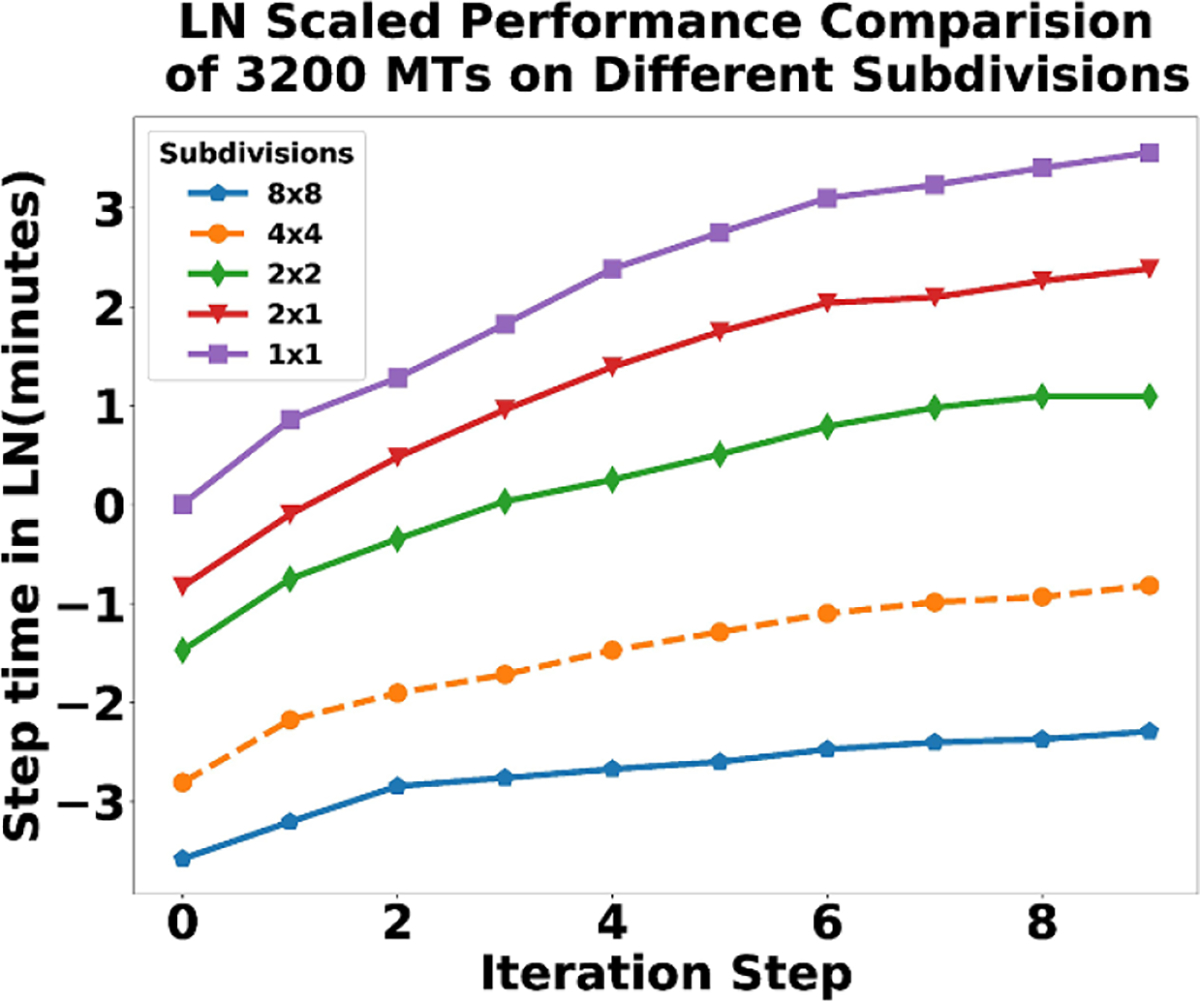
Natural log scaled plot of performance analysis of five separate simulation runs for 10 iterations.

We ran the simulation five times, once with no subdivisions and four times with different subdivisions, as seen in figures 20 and 21. The first run, with no subdivisions, is the 1 × 1 domain. The 1 × 1 case does not use operator splitting by geocell dimension and is equivalent to the Exact Hybrid ODE SSA in algorithm 1.

For each step of $\tau_{\text{ref,}}$ the maximum time step that can be achieved is the adaptive step, $max\{\frac{1}{\text{reactions}},\frac{v_{max}}{l_{max}}\}$. If we move beyond this step size, the ODE solver may miss reaction dynamics. As can be seen in figure 20, the exact algorithm is prohibitively slow because it must take more steps to solve the system. The step time of iteration 10 for the 1 × 1 subdivision in figure 20 reflects the slowdown and takes over 2000 s or approximately 33 min on a single core of an Intel Core i7-7700HQ CPU @ 2.80 GHz. Clearly, this is not practical for long term simulations with serial computation and server grade CPUs would not fare significantly better. In parallel computation, similar bottlenecks for individual geocells would show up if the experiment was scaled up so that each of the 8 × 8 subdivisions was the same size as a 1 × 1 subdivision; however, there would still be the benefit of running parallel computations.

The subdivisions 2 × 1, 2 × 2, 4 × 4, 8 × 8 each show a significant speedup over the original 1 × 1 Exact SSA. Each of these runs uses algorithm 2. In the 2 × 1 case, we see a speedup(caused by subdividing the domain) of around a factor of four instead of a factor of two. The difference may be because larger steps can be taken and the search space is smaller. In the 2 × 2 case, it becomes a factor of twenty. In figure 20 the 8 × 8 and 4 × 4 case look similar due to the time scale, however there is also a significant speedup. There may be diminishing returns to scale beyond 8 × 8, for our initial condition of 3200 MTs. We include the semi-log plot (figure 21) of the same data in figure 20 to make the step time differences more clear.

From these results, we find that the approximate algorithm achieves a significant speedup over the exact algorithm. The resulting speedup comes with a few trade-offs. First, we get the speedup at the potential cost of accuracy due to reactions firing out of order. Second, there is a saturation speedup point for every system. Simulation cells can only be minimized to a factor of the ‘fall off’ distance and still need to maintain well-separatedness. Finally, the practical lower limit of the ODE solver step size depends on the dynamics being simulated. Whereas simulation speed is limited by cell size and ODE solving step size, there is no such limit on scalability—making this algorithm appropriate for modeling very large systems, or smaller systems in greater detail.

## 4. Conclusion and future work

DGGs can be used to simulate complex biological systems using a simulation algorithm derived from their corresponding master equation. We have introduced an approximate algorithm, for spatially embedded and local DGG dynamics, which achieves improvements in performance over an exact algorithm at some potential cost of accuracy. We demonstrated the speedup in simulated dynamics of a DGG model of a plant CMA, which forms a cytoskeletal network and can exhibit localized alignment.

In future work, we plan to run further experiments using this model with many different parameter settings in pursuit of plant science questions. We also plan to experiment with grammars developed to model actin dynamics in neurons. A revised version of the simulator is in development to add new features and to improve performance. The approximate algorithm (Algorithm 2), is highly parallelizable, so we are in the process of implementing a parallel version of the approximate algorithm. Another planned feature is a translator capable of generating model specific code for different types of grammar rules and systems—a front-end for a spatial specific version of the DGG modeling language. There is also potential to reduce the model using machine learning, as in [52] or [53].

## Supplementary Material

MedwedeffMjolsnessSupplemental

## Data Availability

The data that support the findings of this study are openly available at the following URL/DOI: https://github.com/emedwede/CajeteCMA.
