# Supplementary material for: Approximate simulation of cortical microtubule models using dynamical graph grammars
Source: Phys Biol. Author manuscript; Available in PMC 2024 Jul 1. (PMC11216692; doi:10.1088/1478-3975/acdbfb)
Supplement: MedwedeffMjolsnessSupplemental [file NIHMS1921962-supplement-MedwedeffMjolsnessSupplemental.pdf]

Supplementary Material for Approximate Simulation of Cortical Microtubule Models using Dynamical Graph Grammars

Eric Medwedeff[1,2] and Eric Mjolsness[2,3]

[1] Computational Science Research Center, San Diego State University
[2]Department of Computer Science, UC Irvine
[3]Department of Mathematics, UC Irvine

1 Details on the CMA DGG

1.1 Overview and Definitions

There are two different classes of rules, *continuous* and *discrete*. A continuous rule is a rule that updates parameters by solving an ordinary differential equation (ODE). A discrete rule is a rule that rewrites the system graph and may modify parameters by sampling new ones from a factored propensity function. In the main text, we discussed the factorized form of rules and the general form for rules. The discrete rules for this grammar are written in a more compact shorthand form. Newly sampled output values and variables used in rate calculations are sub-parts of a *where* clause. Parameters are exactly sampled to be those values by using a Dirac delta distribution. As stated in the results section, artificial parameters are chosen to evaluate the simulation algorithm and code, rather than ones chosen to represent biophysical knowledge.

Graph Symbol	Type Name
●	growing
○	intermediate
■	retraction
▲	zipper
⧫	junction

Table 1: Graph Node Type Symbol Table

Table 1 lists the symbols used to represent nodes types in the DGG CMA rules. Table 2 lists the commonly used rule parameters in the grammar, and includes a brief description of their meaning. In table 3 we do the same, but for model parameters. Finally, table 4 lists commonly used functions and a description as well.

Rule Parameter	Description
x_n	a point in $\mathbb{R}^2$
u_n	a unit vector in $\mathbb{R}^2$
L	current length of the MT segment
β	minimum distance from collision point
I	an intersection point

Table 2: Rule Parameter Definitions

Model Parameter	Descpription	Value
L_{div}	the maximal dividing length of a segment	1.2 units
L_{min}	the minimal length of a segment	0.125 units
$[Y_g]$	tubulin concentration	1.0 units
v_{plus}	growth rate	1.0 units per time
v_{minus}	retraction rate	0.25 units per time
γ	parametric line parameter	1.0
ϵ	maximal reaction radius	$2.0 * L_{div}$
θ_{crit}	critical angle	$\frac{2\pi}{9}$ radians
c	a constant factor	1.0
k	sigmoid factor	10
$\hat{\rho}_{\text{retract}\leftarrow\text{growth}}$	retraction to growth conversion rate	0.01
$\hat{\rho}_{\text{growth}\leftarrow\text{retract}}$	growth to retraction conversion rate	0.01

Table 3: Model Parameter Definitions

Function	Description
$\hat{\rho}_{\text{grow}}(x) = (x)(v_{plus})$	growth rate function
$\hat{\rho}_{\text{retract}}(x) = (x)(v_{minus})$	retraction rate function
$\hat{\rho}_{\text{zipper}}(x) = x$	zipper rate function
$\hat{\rho}_{\text{junction}}(x) = x$	junction rate function
$\sigma(x;k) \equiv \frac{1}{1+e^{-kx}}$	sigmoid function
$H(x;a) = \begin{cases} 1 & x \geq a \\ 0 & x < a \end{cases}$	unit step function
$\Theta(\text{stmt}) = \begin{cases} 1 & \text{if stmt is true} \\ 0 & \text{if stmt is false} \end{cases}$	indication function

Table 4: Function Descriptions

1.2 Minimal CMA DGG

The cortical microtubule array (CMA) grammar presented is a minimal set of rules capable of leading to the long-time behavior of network formation. The grammar consists of six rules: two for growth, two for retraction, a collision rule with three different outcomes, and a reversible state change of growing to retracting. We introduce and briefly discuss these in the following subsections.

1.3 Positive MT Growth

The positive growth rule models polymerization and effectively elongates the MT. The rule in equation 1 describes MT growth (polymerization), and is a continuous rule. The position of x_2 is updated by solving an ordinary differential equation limited by the dividing length in the direction of the growing node. The function $\hat{\rho}_{\text{grow}}$ (table 4) can be any function of tubulin concentration, but is set to be a linear function with a constant rate of growth v_{plus} and $[Y_g]$ both of which are set in table 3.

$$
\begin{aligned}
&(\ \bigcirc_1 - \bullet_2) \langle\!\langle (\boldsymbol{x}_1, \boldsymbol{u}_1)(\boldsymbol{x}_2, \boldsymbol{u}_2) \rangle\!\rangle \\
&\longrightarrow (\bigcirc_1 - \bullet_2) \langle\!\langle (\boldsymbol{x}_1, \boldsymbol{u}_1), (\boldsymbol{x}_2 + d\boldsymbol{x}_2, \boldsymbol{u}_2) \rangle\!\rangle \\
&\quad \textbf{solving} \quad d\boldsymbol{x}_2/dt = \hat{\rho}_{\text{grow}}([Y_{\text{g}}])(1 - L/L_{\text{div}})\boldsymbol{u}_2
\end{aligned} \tag{1}
$$

1.4 Positive MT Overgrowth

Equation 2 is a discrete rule. When a MT segment becomes long enough, a new node is added in between the previous two nodes and splits the edge. This process is called "overgrowth" with respect to the CMA DGG. A sigmoid function provides a fully activated rate when a dividing threshold has been reached. The new node, $\bigcirc_3$, is placed at a point controlled by the γ parameter on the line between x_1 and x_2. An additional constant c can be used to further scale the rate, but for these simulations it is set to unity.

$$
\begin{aligned}
&(\ \bigcirc_1 - \bullet_2) \langle\!\langle (\boldsymbol{x}_1, \boldsymbol{u}_1), (\boldsymbol{x}_2, \boldsymbol{u}_2) \rangle\!\rangle \\
&\longrightarrow (\ \bigcirc_1 - \bigcirc_3 - \bullet_2) \langle\!\langle (\boldsymbol{x}_1, \boldsymbol{u}_1), (\boldsymbol{x}_2, \boldsymbol{u}_2), (\boldsymbol{x}_3, \boldsymbol{u}_3) \rangle\!\rangle \\
&\quad \textbf{with} \quad c \times \sigma(\frac{\|\boldsymbol{x}_2 - \boldsymbol{x}_1\|}{L_{\text{div}}}; k = 10) \\
&\quad \textbf{where} \quad \begin{cases} \boldsymbol{x}_3 = \boldsymbol{x}_2 - (\boldsymbol{x}_2 - \boldsymbol{x}_1)\gamma \\ \boldsymbol{u}_3 = \frac{\boldsymbol{x}_3 - \boldsymbol{x}_2}{\|\boldsymbol{x}_3 - \boldsymbol{x}_2\|} \end{cases}
\end{aligned} \tag{2}
$$

1.5 Negative MT Retraction

Negative MT retraction is a continuous rule used to model depolymerization. Equation 3 is similar to the growth rule in 1, but instead functions to limit how much the MT segment can retract. The function $\hat{\rho}_{\text{retract}}$ (table 4) can be any function of tubulin concentration, but is set to be a linear function with a constant rate of growth $v_{retract}$ and $[Y_g]$ both of which are set in table 3. The limit, L_{min}, is primarily added to prevent instability from round off errors in the ODE solver caused by two points being too close to one another.

$$
\begin{aligned}
&(\ \blacksquare_1 - \bigcirc_2) \langle\!\langle (\boldsymbol{x}_1, \boldsymbol{u}_1), (\boldsymbol{x}_2, \boldsymbol{u}_2) \rangle\!\rangle \\
&\longrightarrow (\ \blacksquare_1 - \bigcirc_2) \langle\!\langle (\boldsymbol{x}_1 + d\boldsymbol{x}_1, \boldsymbol{u}_1), (\boldsymbol{x}_2, \boldsymbol{u}_2) \rangle\!\rangle \\
&\quad \textbf{solving} \quad d\boldsymbol{x}_1/dt = \hat{\rho}_{\text{retract}}([Y_{\text{g}}])(L/L_{\text{min}}\ )\boldsymbol{u}_1
\end{aligned} \tag{3}
$$

1.6 Negative MT Undergrowth

The rule in equation 4 is a discrete rule used to shorten a MT. In the context of the CMA DGG, the process of shortening is "undergrowth". The rule in equation 2 inserts more intermediate nodes into the system and the negative MT undergrowth rule removes them. Here, a Heaviside function H scaled by a constant c set to unity is used. When the retraction node gets close enough to its intermediate neighbor, the rate function activates. If this rule is selected to occur, the neighboring intermediate node is removed.

$$
\begin{aligned}
&(\ \blacksquare_1 - \bigcirc_2 - \bigcirc_3)\,\langle\langle(\boldsymbol{x}_1,\boldsymbol{u}_1),(\boldsymbol{x}_2,\boldsymbol{u}_2),(\boldsymbol{x}_3,\boldsymbol{u}_3)\rangle\rangle \\
&\longrightarrow (\ \blacksquare_1 - \bigcirc_3)\,\langle\langle(\boldsymbol{x}_1,\boldsymbol{u}_1),\varnothing,(\boldsymbol{x}_3,\boldsymbol{u}_3)\rangle\rangle \\
&\qquad \textbf{with} \quad c \times H(-\|\boldsymbol{x}_2-\boldsymbol{x}_1\|; -0.2 * L_{\text{div}})
\end{aligned} \tag{4}
$$

1.7 MT Collision

The collision rule is more combinatorially complex than the rest of the rules, having three alternative discrete outcomes. The LHS of the grammar rule can be seen in equation 5. It represents the case where a growing end of a MT is close enough to the interior of another MT.

$$
\begin{pmatrix} & \bigcirc_1 - \bigcirc_2 - \bigcirc_3 \\ \bigcirc_4 - \bullet_5 & \end{pmatrix} \begin{aligned} &\langle\langle(\boldsymbol{x}_1,\boldsymbol{u}_1),(\boldsymbol{x}_2,\boldsymbol{u}_3),(\boldsymbol{x}_3,\boldsymbol{u}_3), \\ &\quad (\boldsymbol{x}_4,\boldsymbol{u}_4),(\boldsymbol{x}_5,\boldsymbol{u}_5)\rangle\rangle \end{aligned} \tag{5}
$$

Each of the three discrete outcomes makes use of locally defined rule specific parameters from the where clause in equation 6. Normally, the where clause would follow the with clause like in equation 8, but it is separated out for clarity. In equation 6, I_1, I_2 are intersection points of lines parameterized by $\alpha_1, \alpha_2 \in (-\infty, \infty)$ starting at the point x_2 in the directions u_1, u_2. The parameter α_1 indicates where the growing edge $(\bigcirc_1, \bullet_5)$ intersects edge $(\bigcirc_1, \bigcirc_2)$ and α_2 indicates where the growing edge $(\bigcirc_1, \bullet_5)$ intersects edge $(\bigcirc_2, \bigcirc_3)$.

$$
\textbf{where} \quad \begin{cases}
\alpha_1 = (\boldsymbol{x}_5 \cdot (\boldsymbol{x}_1 - \boldsymbol{x}_2) - \boldsymbol{x}_2 \cdot (\boldsymbol{x}_1 - \boldsymbol{x}_2))/||\boldsymbol{x}_1 - \boldsymbol{x}_2||^2 \\
\alpha_2 = (\boldsymbol{x}_5 \cdot (\boldsymbol{x}_3 - \boldsymbol{x}_2) - \boldsymbol{x}_2 \cdot (\boldsymbol{x}_3 - \boldsymbol{x}_2))/||\boldsymbol{x}_3 - \boldsymbol{x}_2||^2 \\
I_1 = \boldsymbol{x}_2 + \alpha_1(\boldsymbol{x}_1 - \boldsymbol{x}_2) \\
I_2 = \boldsymbol{x}_2 + \alpha_2(\boldsymbol{x}_3 - \boldsymbol{x}_2) \\
\beta_1(\alpha_1) = \begin{cases} ||\boldsymbol{x}_5 - I_1||^2, & 0 < \alpha_1 < 1 \\ ||\boldsymbol{x}_5 - \boldsymbol{x}_1||^2, & \alpha_1 \geq 1 \\ ||\boldsymbol{x}_5 - \boldsymbol{x}_2||^2, & 0 \leq \alpha_1 \end{cases} \\
\beta_2(\alpha_2) = \begin{cases} ||\boldsymbol{x}_5 - I_2||^2, & 0 < \alpha_2 < 1 \\ ||\boldsymbol{x}_5 - \boldsymbol{x}_3||^2, & \alpha_2 \geq 1 \\ ||\boldsymbol{x}_5 - \boldsymbol{x}_2||^2, & 0 \leq \alpha_2 \end{cases} \\
\beta = min(\beta_1, \beta_2)
\end{cases} \tag{6}
$$

The functions β_1, β_2 are used to calculate the minimum distance to intersection along the respective segments. Since the growing edge $(\bigcirc_1, \bullet_5)$ could intersect $(\bigcirc_1, \bigcirc_2)$ or $(\bigcirc_2, \bigcirc_3)$, we must find the closest of the two. Hence, we use $\beta = min(\beta_1, \beta_2)$.

The first discrete outcome is zippering (equation 7), with additional parameters defined in the where clause in equation 6. The rate function primarily depends on the incoming MT being at a critical angle and the incoming MT being on a collision trajectory with another MT in the reaction radius. If these conditions are met, the rate function becomes non-zero and increases as the collision gap narrows. The rewrite on the right hand side (RHS) attaches the growing end to the segment and transforms it into a zippering node.

$\longrightarrow$ 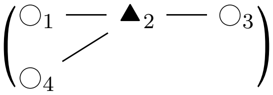 $\langle\!\langle (\boldsymbol{x}_1, \boldsymbol{u}_1), (\boldsymbol{x}_2, \boldsymbol{u}_2), (\boldsymbol{x}_3, \boldsymbol{u}_3), (\boldsymbol{x}_4, \boldsymbol{u}_4), \varnothing \rangle\!\rangle$

$$\textbf{with} \quad \hat{\rho}_{\text{zipper}}(|\boldsymbol{u}_2 \cdot \boldsymbol{u}_4|/|\cos(\theta_{\text{crit}})|) \times \exp(-\beta^2/2\epsilon^2) \times (\Theta(0 < \alpha_1 < 1) + \Theta(0 < \alpha_2 < 1) + \Theta(\alpha_1 = \alpha_2))$$

$$\textbf{where} \quad \left\{ \boldsymbol{u}_4 = \frac{\boldsymbol{x}_2 - \boldsymbol{x}_4}{\|\boldsymbol{x}_2 - \boldsymbol{x}_4\|} \right. \tag{7}$$

The second case is junction formation (equation 8), with additional parameters defined in the where clause in equation 6. The rate function, like zippering, depends on the critical angle and the collision trajectory. If the conditions are met, the propensity increases and a junction is formed if the rule is selected to occur. The rewrite effectively crosses the growing MT over the intermediate MT segment.

$\longrightarrow$ 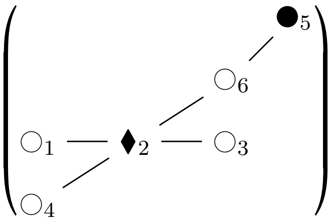 $\langle\!\langle (\boldsymbol{x}_1, \boldsymbol{u}_1), (\boldsymbol{x}_2, \boldsymbol{u}_2), (\boldsymbol{x}_3, \boldsymbol{u}_3), (\boldsymbol{x}_4, \boldsymbol{u}_4), (\boldsymbol{x}_5, \boldsymbol{u}_4), (\boldsymbol{x}_6, \boldsymbol{u}_4) \rangle\!\rangle$

$$\textbf{with} \quad \hat{\rho}_{\text{junction}}(|\boldsymbol{u}_2 \cdot \boldsymbol{u}_4|/|\cos(\theta_{\text{crit}})|) \times \exp(-\beta^2/2\epsilon^2) \times (\Theta(0 < \alpha_1 < 1) + \Theta(0 < \alpha_2 < 1) + \Theta(\alpha_1 = \alpha_2))$$

$$\textbf{where} \quad \begin{cases} \boldsymbol{u}_4 = \frac{\boldsymbol{x}_2 - \boldsymbol{x}_4}{\|\boldsymbol{x}_2 - \boldsymbol{x}_4\|} \\ \boldsymbol{x}_6 = \boldsymbol{x}_2 + \frac{L_{max}}{4} \boldsymbol{u}_4 \\ \boldsymbol{x}_5 = \boldsymbol{x}_2 + \frac{L_{max}}{2} \boldsymbol{u}_4 \end{cases} \tag{8}$$

The third discrete outcome is catastrophe (equation 9), with additional parameters defined in the where clause in equation 6. In this case, we have the propensity increasing as long as the MT is on a collision course with the nearby MT segment. The rewrite causes the incoming MT to change from a growing to a retracting state, simulating catastrophe.

$\longrightarrow$ 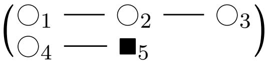 $\langle\!\langle (\boldsymbol{x}_1, \boldsymbol{u}_1), (\boldsymbol{x}_2, \boldsymbol{u}_3), (\boldsymbol{x}_3, \boldsymbol{u}_3), (\boldsymbol{x}_4, \boldsymbol{u}_4), (\boldsymbol{x}_5, \boldsymbol{u}_5) \rangle\!\rangle$

$$\textbf{with} \quad c \times \exp(-\beta^2/2\epsilon^2) \times (\Theta(0 < \alpha_1 < 1) + \Theta(0 < \alpha_2 < 1) + \Theta(\alpha_1 = \alpha_2)) \tag{9}$$

1.8 State Changes

The state change rule in equation 10 is a bi-directional discrete rule. Growing ends become retracting ends at a rate of $\hat{\rho}_{\text{retract}\leftarrow\text{growth}}$. Retracting ends become growing ends at a rate of $\hat{\rho}_{\text{growth}\leftarrow\text{retract}}$. These rates are typically chosen to be near zero, so that they occur less frequently than the other discrete rules [1]. More rates can be found in [2].

$$
\begin{aligned}
&(\bullet_1)\,\langle\!\langle \boldsymbol{x}_1, \boldsymbol{u}_1 \rangle\!\rangle \quad \longleftrightarrow \quad (\blacksquare_1)\,\langle\!\langle \boldsymbol{x}_1, \boldsymbol{u}_1 \rangle\!\rangle \\
&\quad \textbf{with} \;\; (\hat{\rho}_{\text{retract}\leftarrow\text{growth}}, \hat{\rho}_{\text{growth}\leftarrow\text{retract}})
\end{aligned}
\tag{10}
$$

2 Calculation of correlation length difference z-score

The first and second experiments produced angle correlation lengths as detailed in the main text of $\xi_1 \pm \sigma_{‘} = 1.34 \pm 0.039$ and $\xi_2 \pm \sigma_2 = 3.14 \pm 0.06$. We estimate the z-score for the difference $\xi_2 - \xi_1$ as

$$
Z = \frac{\xi_2 - \xi_1}{\sigma_2 + \sigma_1} \simeq \frac{3.14 - 1.34}{0.06 + 0.039} \simeq 18.2
$$

The two experiments resulted in different numbers of microtubule segments. If we zoom in on the first experiment window by a factor of 1.6 this population becomes equal, although other parameters of the model become different. So a very generous interpretation of ξ_1 is to multiply it by 1.6 yielding $\hat{\xi}_1 \pm \hat{\sigma}_1 = 1.34 * 1.6 \pm 0.039 * 1.6$ and a z-score of

$$
\hat{Z} = \frac{\xi_2 - \hat{\xi}_1}{\sigma_2 + \hat{\sigma}_1} \simeq \frac{3.14 - 1.34 * 1.6}{0.06 + 0.039 * 1.6} \simeq 8.14
$$

This is larger than the $Z = 5$ standard deviations sometimes used for high certainty because the corresponding p-value for a Gaussian distribution is roughly 3×10^{-7}. Such p-values are $\ll 0.05$, a commonly used threshold for "statistical significance". Of course, we may not have a Gaussian distribution. The worst case distributions however must still obey the Chebyshev inequality under which the p-value must be at most $1/Z^2 \simeq .003 \ll .05$ or even $1/\hat{Z}^2 \simeq .015 \ll .05$, in an extremely conservative calculation.

References

[1] Wightman R, Turner SR. Severing at sites of microtubule crossover contributes to microtubule alignment in cortical arrays. The Plant Journal. 2007;52(4):742–751.

[2] Shaw SL, Kamyar R, Ehrhardt DW. Sustained Microtubule Treadmilling in Arabidopsis Cortical Arrays. Science. 2003 06;300(5626):1715–1718.
